# Biological effects of the hypomagnetic field: An analytical review of experiments and theories

**DOI:** 10.1371/journal.pone.0179340

**Published:** 2017-06-27

**Authors:** Vladimir N. Binhi, Frank S. Prato

**Affiliations:** 1 A.M. Prokhorov General Physics Institute, Moscow, Russia; 2 M.V. Lomonosov Moscow State University, Moscow, Russia; 3 Lawson Health Research Institute, Ontario, Canada; 4 University of Western Ontario, Ontario, Canada; Syddansk Universitet, DENMARK

## Abstract

During interplanetary flights in the near future, a human organism will be exposed to prolonged periods of a hypomagnetic field that is 10,000 times weaker than that of Earth’s. Attenuation of the geomagnetic field occurs in buildings with steel walls and in buildings with steel reinforcement. It cannot be ruled out also that a zero magnetic field might be interesting in biomedical studies and therapy. Further research in the area of hypomagnetic field effects, as shown in this article, is capable of shedding light on a fundamental problem in biophysics—the problem of primary magnetoreception. This review contains, currently, the most extensive bibliography on the biological effects of hypomagnetic field. This includes both a review of known experimental results and the putative mechanisms of magnetoreception and their explanatory power with respect to the hypomagnetic field effects. We show that the measured correlations of the HMF effect with HMF magnitude and inhomogeneity and type and duration of exposure are statistically absent. This suggests that there is no general biophysical MF target similar for different organisms. This also suggests that magnetoreception is not necessarily associated with evolutionary developed specific magnetoreceptors in migrating animals and magnetotactic bacteria. Independently, there is nonspecific magnetoreception that is common for all organisms, manifests itself in very different biological observables as mostly random reactions, and is a result of MF interaction with magnetic moments at a physical level—moments that are present everywhere in macromolecules and proteins and can sometimes transfer the magnetic signal at the level of downstream biochemical events. The corresponding universal mechanism of magnetoreception that has been given further theoretical analysis allows one to determine the parameters of magnetic moments involved in magnetoreception—their gyromagnetic ratio and thermal relaxation time—and so to better understand the nature of MF targets in organisms.

## Introduction

It is known that the weak magnetic field (MF) can trigger a variety of biological responses. MF action can change the concentration of different substances and signaling molecules—this is the area of research primarily in laboratory magnetobiology, e.g. [1–4]. The geomagnetic field affects the everyday behavior of some animals, e.g. [5, 6]. It is also used as a navigational cue by many animals in long seasonal migrations, e.g. [7]. Background MFs of commercial frequencies sometimes correlate with the incidence of some forms of cancer [8] and other pathologies [9]. The electromagnetic (EM) environment is a subject of research in the epidemiology of electromagnetic fields, e.g. [10]. It is standardized by safety standards, e.g. [11] and is accounted for in animal behavior studies [12]. Finally, heliobiology investigates the correlation of the geomagnetic disturbance to the health of humans and other biospheric processes, e.g. [13–15], the MF being regarded as an immediate cause of the correlations [16]. All these effects form a variety of the so-called non-thermal magnetic biological effects.

Despite the abundance and variety of empirical data, the mechanisms underlying magnetoreception, although speculated on extensively [17, 18], are still not identified.

The non-thermal effects can appropriately be categorized into two groups: specific and nonspecific effects. The first group includes the MF reactions of seasonally migratory animals that have formed the so-called magnetic sense, in the course of evolution. It is a perfect and not yet fully understood mechanism that allows animals to sense MF changes of a few to tens of nT, e.g. [19]. This group has sufficient reproducibility, according to the scientific standards; however it is not this group that is the focus of the current study. Effects of the second group—nonspecific reactions—are quite widely represented both by the variety of biological species of the sensitive organisms and by the diversity of magnetic-dependent characteristics. These nonspecific effects form the bulk of publications in magnetobiology, numbering now in the tens of thousands. It is these effects that, due to their generality, are the object here of our attention.

A distinctive feature of the nonspecific reactions is their poor reproducibility; almost all the studies in this area are unique. One of the principal causes for poor reproducibility is the random character and unpredictability of the nonspecific non-thermal effects. It has not yet been possible to find the causes that control the appearance of nonspecific effects or summarize the outcomes of this research under any theory [16]. In these conditions, the mere accumulation of low-reproducible experimental data has failed to elicit an understanding of the biophysical underpinning of these effects and will hardly be useful in disclosing the nature of magnetoreception.

As this article shows, among the variety of nonspecific magnetic biological phenomena, the effects of the hypomagnetic field occupy a special place because of their higher reproducibility and the fact that, as shown here, the comparison of experiment and theory is able to give more information about the MF targets in organisms.

We have used the term “MF target” rather than “MF receptor” extensively. The term “receptor” implies that there is a specific receptor—a biophysical construct that is capable of reacting to MF and transferring the signal to the biological level. An example is the magnetosome chain in magnetotactic bacteria, e.g. [20], or the magnetosensitive retina in the eyes of some birds, e.g. [21]. This is not the case in nonspecific magnetoreception, where biological magnetic effects exist, while specific magnetoreceptors do not, e.g., in *E. coli* cells and other species that have not developed through evolutionary pressures specific magnetic receptors [22]. In this case we are talking about MF targets that are potentially different over different organisms and even over individuals. Further, known proteins and parts of macromolecules can presumably be MF targets depending on local conditions. As well, we have been careful to distinguish between a primary physical target, i.e., magnetic moments, and a biophysical MF target that contains the moments and can produce a biophysical or biochemical signal depending on the state of these moments. These signals may not have a definite transduction path and elicit a biological reaction mostly by chance. A clear example of the MF biophysical target is a flavin–tryptophan radical pair, e.g. [23], while the pair of electron spin magnetic moments inside is a physical primary MF target. However it is not always the case that an MF-induced change in the state of physical MF targets result in the biophysical construct producing a potentially observable signal.

In the literature, various terms have been used to describe the conditions under which the value of the local MF is considerably reduced. These terms are magnetic vacuum, hypomagnetic field, hypogeomagnetic field, magnetic deprivation, zero magnetic field, near-zero field, etc. In this article, we use mostly the term “hypomagnetic field” (HMF) that implies a significant local MF weakening in relation to the geomagnetic field *H*_g_ ∼ 50 *μ*T. In a hypomagnetic field *H*(*t*) ≡ *H* + *h*(*t*) the following inequalities are true simultaneously,

$$H\ll H_{g},\;\;\bar{h}\ll H_{g},\;\;\bar{dh/dt}\ll h^{\prime },$$ (1)

where *H* is the value of the constant component of the local MF, $\bar{h}$ is the rms value of its variable component, *h*′ is a certain threshold for the rms rate of the MF changes. The latter inequality guarantees the absence of the effects of induced electric fields. In experiments with HMF, usual values of *h*′ satisfy this inequality with a large excess.

Because of fluctuations in the geomagnetic field *H*_g_, the definition of HMF is not absolute and always relates to the time interval *T* of observation. An MF with fixed values of *H*, $\bar{h}$, and $\bar{dh/dt}$ can be an HMF at some intervals that do not exceed *T*, while not being such at longer intervals due to the involvement of the geomagnetic variations in *H*_g_.

The terrestrial magnetic field is a factor in biological evolution. Approximately every 200 thousand years, the Earth’s magnetic polarity changes its sign, and during the reversal, living organisms find themselves in a significantly reduced MF for periods of millennia. It makes no sense to discuss the biological role of this quasiperiodic exposure to the HMF because of a much more dangerous synchronous factor—ionizing solar radiation which normally is repelled away by the geomagnetic field. However, HMF effects are of relevant current importance.

Biological effects of HMFs are interesting in that organisms on Earth might have adapted to the presence of the geomagnetic field in a billion-year evolution. Elimination of the geomagnetic field may affect organisms. Hypomagnetic field has been suggested to influence negatively on the early development of organisms, e.g. [24] and functioning of the central nervous system, e.g. [25], although positive effects are also possible, e.g. [26]. Besides the practical importance, hypomagnetic effects are also of fundamental importance as their physical origin remains unclear. Hence, here a significant theoretical effort has been directed toward a general understanding of these effects.

It is usually believed that primary physical mechanisms of magnetoreception are identical for exposure to geomagnetic field (GMF), ac/dc ELF MFs of the same level, and HMF.

Only two types of the primary mechanisms are discussed in the studies of magnetic orientation in animals. One considers the chemical reactions involving spin-correlated biradicals, e.g. [27] that occur in the ordered cryptochrome arrays in eye retina. The other is based on the dynamics of magnetic nanoparticles naturally present in the body, e.g. [28, 29]. A synergism of these mechanisms has been also proposed in [30, 31] and developed in [32]. However, these two types of mechanisms do not make a complete list, since the magnetic effects exist also in non-magnetotactic bacteria, e.g. [33] and cell cultures, see for review in [34] where, likely, neither ordered cryptochromes nor magnetic nanoparticles are present. Furthermore, in laboratory magnetobiology, observed are magnetic effects that, although of low reproducibility, are of a clear nonlinearity and frequency selectivity. These phenomena, for example, amplitude and frequency “windows” in magnetic biological reactions, mostly do not occur in magnetic orientation of animals. Therefore, a search is going on for fundamental molecular processes, or general mechanisms, underlying magnetoreception.

One such mechanism was recently proposed [35, 36]; it considers a non-uniform precession of magnetic moments in the magnetic field. The mechanism links within a single equation the field parameters and the characteristics of MF target—its gyromagnetic factor and thermal relaxation time. The model describes the effects of both ac and dc MFs, including the effect of HMF. One important consequence is that there are greater chances to observe an HMF effect than the effect of any other weak MF in the same body. HMF effect is significantly more likely because HMF conditions affect the dynamics of all precessing magnetic moments irrespective of their physical properties.

The necessary existence of the HMF effect can be illustrated in a simple quantum mechanical way. In a MF *H*, the quantum energy levels of a magnetic moment split into the Zeeman sublevels that are separated by an energy gap of ℏ*γH*, where ℏ is the Plank constant and *γ* is the gyromagnetic ratio. In the zero MF, the splitting is zero, i.e., the sublevels degenerate into a single level. This is a quantum formulation of the classical statement that all magnetic moments that normally precess stop precessing in a zero MF. The levels degenerate provided their width, which is of the order of ℏ/*τ*, where *τ* is the thermal relaxation, or decoherence, time, becomes comparable with the separation of the QM levels. Hence, a critical MF *H* ∼ 1/*γτ* follows that define a value below which some qualitative changes are possible at the quantum level. Since any magnetic effects, including biological ones, start from the MF interaction with magnetic moments, we conclude that those qualitative changes can cause a biological response [18] p. 47.

In this regard, the next question is interesting. What kind of information on the physical characteristics of the MF target in organisms could HMF-related experiments bring? Of course, any such information could only be extracted by comparing the experimental data with a physical theory of magnetoreception. In this article, we consider the extent to which the known putative primary mechanisms are suitable for describing the effects of HMF and identifying the most plausible characteristics of the MF target.

The aim of this study was to 1) summarize the experimental work on the effects of the hypomagnetic field and their possible theoretical explanations, 2) show the special character of the effects of HMFs and their increased potential in revealing the nature of nonspecific magnetic effects, and 3) suggest a methodologically consistent approach of experiments in this area.

## Experimental data

One of the early works on the biological effects of the hypomagnetic field was a study with a few subjects [37] undertaken in anticipation of the Apollo mission to the moon. It was found that a 10-day stay in the conditions of an HMF less than 50 nT caused approximately 20% decrease in the critical scotopic flicker-fusion frequency in three of four participants. At the same time, all the other investigated physiological responses remained unchanged [38]. In early 1960s, several studies did not find HMF effects on the development of chicken embryo and cultures of mammalian tissue [39]. However, in subsequent years, various effects of exposure to HMF have been found in mammals, snails, insects, plants, and other biological systems.

The number of original experimental reports in the area of HMF effects is somewhat more than 200, with nearly half being published in the last 10 years. Part of them are summarized in reviews [40–45] and [18] p. 43–47. Information about old publications that are difficult to obtain can be found in [39, 46, 47] and in [48] p.112–125. All these reviews contain analyses in terms of biology and include between 10–30 publications. Here we have put together a much more extensive review referencing more than a hundred papers, while analyzing both biological and physical aspects. The latter includes field values, duration of exposure and the type and size of the exposure systems of each publication. Unfortunately, some 80 papers in Russian, English, Chinese, French, and Ukrainian dated 1962–2015 have not been available to us, and are therefore not included in this review; the list of these articles is available as supporting information “S1 References.” Specifically, the method of article selection and data extraction and analysis is given below.

### Methods

#### Article selection

The subject of the search, which is called here “HMF effect,” is expressed in literature in many different forms, which means that scientific terminology in this area is still evolving. A targeted search for relevant publications on the HMF biological effect was, for this reason, a challenge.

First, we used a scientific information aggregator The Global Science Gateway, or WorldWideScience [worldwidescience.org], to select papers that contained “hypomagnetic” in their titles. The aggregator has an access to 106 databases all over the world. This gave, after a subsequent semantic control, 14 articles that included original results suitable for further investigation. At this stage and later, we took into account only peer-reviewed articles and did not restrict the date range of documents.

PubMed database of the National Center for Biotechnology Information [pubmed.gov] provides an advanced search capability for word combinations. We have used “zero magnetic,” “null magnetic,” “magnetic deprivation,” “magnetic field compensation,” and “magnetic shielding.” This gave about 40 more articles.

Separately, we have studied the contents of a specialized scientific journal, Bioelectromagnetics, and found a few articles that could not be selected based on keywords, because the HMF effect was not their main subject.

Relevant papers in Russian have been found in the open bibliographic database of Russian scientific periodicals eLIBRARY [elibrary.ru]. As a rule, articles in Russian include an abstract in English. The same keywords as above have been used therefore, which resulted in about 20 items in the domain of biology. We also reviewed the contents of “Biofizika,” a specialized journal, for the past five years, and separated about 10 more articles.

Further search for the relevant articles both in English and in other languages continued through examination of the lists of references in each of the articles that had been identified. Many articles that contain results on the HMF effect are problematic for the targeted search since their titles, as for example “Reduction of the background magnetic field …” and “… geomagnetic field screening,” are related to the subject of search only by their meaning. For this reason, examining the lists of references of the already selected papers was most productive. This type of search has been repeated with every new relevant article iteratively until no new articles could be detected. This gave us more than 160 additional references that referred both to the original experimental data and to reviews, collections, and books.

On completion of the search, we had the list of about 250 bibliographic sources, of which about 80 were not available to us primarily because of the lack of a resource in the local library and its absence in the Internet. Nearly 170 full-text items have been carefully studied. However about 30 of them have been found unsuitable for further analysis, because they did pass one or more of the exclusion criteria listed below. Finally, 137 articles are used for further numerical analysis.

In selecting these articles, we thus applied one inclusion criterion: The text contains information about a biological effects of weak MF that is considerably less than the GMF. Next, four qualitative exclusion criteria were used: (i) The text is an abstract of conference or a non-peer-reviewed article. (ii) The text is a review with no original data. (iii) The text of article does not contain quantitative information on the MF magnitude of the exposure. (iv) The numerical results on the HMF effect in the article have been published by these authors in their other article that has already been considered relevant.

We did not assess the quality of experimental work and this was not an exclusion criterion. Instead, we addressed any deficiency in the completeness of the description of experiments in Table 1.

**Table 1:** Data on the effects of hypomagnetic field.

Object	Property	Measurand	E, %	Type	HMF	Time	Size	Ques.	Ref.
Chinese hamster HeLa cells	Growth rate	Cell count	2.7**	?	100	4 d	?	MCS	[49]
Human	Scotopic critical flicker fusion	Fusion frequency	38	⊙	50	10 d	800		[50]
Honeybees	Swarming	Deviations in the waggle dance	100	⊙	2000	1 h	100		[51]
C3H mice	Albumin-induced phagocytic activity	Acid phosphatase activity	77	⊙	80	18 h	360		[39]
House sparrow	Circadian and hopping rhythm	Perch hopping activity	8	⊙	240	112 h	137		[52]
E. coli, etc	Resistance to antibiotics	Staphylococcus resistance to tetracycline	35	□	26	60 d	30	S	[53]
Human fibroblasts and lymphocytes	Conformation of chromatin	Cell lysate viscosity	24	⊙	100	40 m	20		[54]
Pea roots	Cell reproduction	Mitotic index	110	□	54*	72 h	30		[55]
Wheat seedlings	Growth rate	Root and coleoptile length	12	□	70*	72 h	30	MS	[56]
Human embryo fibroblasts	Cell proliferation	Mitotic rate	240	□	0.5	45 h	6	MCS	[48]
Frog Xenopus laevis	Ability of tadpoles to change color	Melanophore index in the tail region	9.1**	⊙	1800	1 h	100	MC	[57]
Rainbow trout Salmo gairdneri	Orientation	Rayleigh statistic	47	□	4000	20 m	?	MC	[58]
Belladonna hairy root culture	Growth rate	Root length	48	□	5	116 h	600		[59]
Corn primary roots	Gravitropic response	Root geometry	37	□	5	12 h	600		[60]
Japanese newt larvae	Developmental abnormalities	Percent abnormal embryos	190	□	5	5 d	?	MS	[24]
Lentil sprouts	Rate of sprout growth	Length of sprouts	51	□	2	6 d	12		[61]
Pea, flax, and lentile roots	Proliferative activity in meristem cells	G1 phase duration	100	□	2	60 h	12	MCS	[62]
Pea roots	RNA and protein synthesis in meristem cells	RNA content	40	□	2	30 h	12	MCS	[63]
Pea seedlings	Features of mitochondrial organelles	Size of organelles	100	□	2	3 d	12	MCS	[64]
Common frog eggs	Fertilized oocyte development	Time to first cleavage	13	⊙	100	120 m	20	S	[65]
Myosin phosphorylation	32P incorporation into myosin light chains	Cherenkov counts	40	□	100*	6 m	30	M	[66]
Guinea pig	Blood biochemistry after X-ray exposure	Epinephrine level	86	□	4500	30 m	?	M	[67]
Newborn rats	Motor activity of ependymal cells	Delay of slowing down	79	⊙	1000	60 m	?	MCS	[68]
Wistar rats	Routin blood analysis	Blood monocytes content	94	⊙	500	4 w	60	S	[69]
Friend erythroleukemia cells	Culture growth cycle	Cell diameter	3.7	□	20	4 d	240	S	[70]
Land snail	Nociception parameters	Latency of response to heat	4.1**	⊙	100	15 m	100		[71]
Cork oak somatic embryos	Germination rate	Percentage of germination	31	⊙	1000	30 d	38		[72]
Budgerigars	Acoustic behavior	Frequency of cries	52	□	15	17 d	230	M	[73]
Pea seedlings	Development rate	Cell elongation	34	□	130	24 h	38		[74]
C57 male mice	Stress-induced analgesia	Latency of response to heat	61	□	4000	2 h	27		[75]
Human blood	Ions in blood serum	Cuprum concentration	22	⊙	500	48 h	60	S	[76]
Human blood	Hemolysis parameters	Rate of hemolysis	180	⊙	500	72 h	60	S	[77]
Pea roots	Developmental characteristics of meristem cells	Phytoferritine content in plastids	89	□	2	3 d	12	MCS	[78]
Hamster	Content of neurotransmitters in brain	GABA content	17	□	100	180 d	70		[79]
Mole-rat brain	Distribution of c-Fos neurons	Density of neurons	82	□	300	1 h	?	M	[80]
CD1 mice	Nociception parameters	Latency of response to heat	41	□	340	2 h	20		[81]
Fruitfly	Eye neural cell activity	Electric retinographic potential	43	⊙	500	20 h	60	MS	[82]
Human T-lymphocite cells	Cytosolic Ca2+ concentration	Indo-1 fluorescence signals	4.5	⊙	300	10 m	80		[83]
Potato Solanum tuberosum	Primary nitrogen metabolism	Nitrogen content	51	□	50	48 h	?	MCS	[84]
E. coli	Resistance to antibiotics	Minimum inhibitory concentration	1200	⊙	500	6 d	60	MS	[85]
Chick embryos	Long-term memory	Avoidance rate	32	⊙	680	21 d	80	S	[86]
Pathogen bacteria	Resistance to antibiotics	Minimum inhibitory concentration	940**	⊙	500	6 d	60	MS	[87]
C57 mice	Formation of spleen cell colonies	Number of colonies	37	□	0.5	3 h	6	MCS	[88]
Drosophila melanogaster	Memory in consequtive generations	Memory index	95	⊙	680	100 d	80		[89]
Pea seedlings	Seedling growth	Epicotyl length	23**	□	2000	4 d	?	M	[90]
Potato	Growth rate	Amount of chlorophyll	130	⊙	500	28 d	60		[91]
CD1 mice	Nociception parameters	Reaction latency to a thermal stimulus	88	□	440	5 h	20		[92]
Human germ cells	Cell properties	Cell velocity	38	⊙	500	18 h	60	MCS	[93]
Friend erythroleukemia cells	Gene expression	Hb RNA labelling	16**	□	20	96 h	240	S	[94]
Friend erythroleukemia cells	DNA replication	The rate of	21**	□	20	96 h	240	S	[95]
Human spermatozoa	Cell behavior	Number of rapid cells	22	⊙	500	3 h	60	MS	[93]
Cress roots	Gravitropic response	Number of roots growing up	210	□	20*	1 h	30	S	[96]
CD1 mice	Nociception parameters	Latency of response to heat	82	□	350	5 h	20		[97]
Planarian Dugesia tigrina	Intensity of fission	Relative number of fissions	79	□	300*	4 h	20		[98]
Planarian Dugesia tigrina	Intensity of fission	Relative number of fissions	15	□	5*	4 h	20		[98]
Golden hamster	Noradrenergic activities in brainstem	Norepinephrine content	54	□	100	180 d	70		[99]
Human blood	Rheological properties of blood	Blood viscosity	4.9	□	75	30 m	120	S	[100]
Frog Rana temporaria	Sciatic nerve excitability	Membrane potential	41	□	170	50 m	18	MS	[101]
Snail Planorbis carinatus	Raman Spectra of neuronal carotenoids	Ratio of spectral peaks	34	□	200	90 m	18	MS	[101]
Planarian Dugesia tigrina	Animal morphology	Regeneration speed	140	□	10000	10 d	200	MCS	[102]
Planarian Dugesia tigrina	Animal morphology	Regeneration speed	19	□	10000	10 d	200	MCS	[103]
Planarian Dugesia tigrina	Intensity of fission	Number of divided animals	210	□	100*	4 h	20		[104]
NMRI mice	Development of embryonic cells	Viability of fibroblasts	96	□	200	48 h	?	S	[105]
Human	Parameters of cognitive processes	Number of errors	7.9	⊙	400	45 m	100		[25]
Wistar rats	Content of elements in hair	Cr content	510	□	20	210 d	?	MC	[106]
Human blood	Blood viscosity dynamics	Correlation with sunspot area	100	□	75	30 m	120	MS	[107]
Magnetospirillum magneticum	Gene expression	mms13 up-regulation	67	⊙	500	16 h	90	S	[108]
Calf brain	Tubilin assembly	Optical absorption at 350 nm	30	⊙	100	20 m	20	S	[109]
Human	Parameters of cognitive processes	Processing time, the number of errors	1.6	⊙	400	45 m	100	S	[110]
Wistar rats	Embryonic abnormalities	Quality changes	100	□	10000	374 h	200	MC	[111]
Land snail Helix albescens	Nociception parameters	Latency of response to heat	27	□	10000	30 h	200	MC	[112]
Planarian Dugesia tigrina	Regeneration parameters	Motion speed	23	□	10000	345 h	200	MCS	[113]
CD1 mice	Nociception parameters	Latency of response to heat	95	□	350	10 h	20		[114]
Wistar rats	Peritoneal macrophages features	NO production after stimulation	33	□	12000	180 d	?	M	[115]
Breeding eggs (Gallus domesticus)	Hatching parameters	Proportion of weak or crippled birds	130	⊙	740	24 d	160	M	[116]
BALB/C mice	Blood properties	Thrombocyte aggregation time	33	□	0.5	2.5 h	6	MCS	[117]
Micromycete U. consortiale	Mycelium growth	Delay in sporulation	240	□⊙	2000	?	11	MCS	[118]
Human erithrocytes	Erythrocytes osmosis stability	Haemoglobin concentration	60	□	0.5	1 h	6	MCS	[119]
Mole-rats	c-Fos expression distribution in brain	Density of c-Fos cells in thalamic nucleus	82	□	300	1 h	?	M	[120]
Rat satellite skeletal muscle cells	Proliferation and differentiation	The number of nuclei in myotubes	100	□	300	7 d	?	MCS	[121]
NMRI mice	Embryogenesis	Birth rate	100	□	200	12 d	20		[122]
Human endothelial cells	Proliferation and gene expression	Number of cells	29	□	500	2 d	?	MS	[123]
Fibrosarcoma HT1080 cells	Cell proliferation	Growth rate	29	⊙	500	4 d	?	M	[124]
Planarian Dugesia tigrina	Infradian rhythms	Motion speed spectrum	24	□	10000	690 h	200	MCS	[125]
Human VH-10 fibroblasts	Mitochondrial net reorganization	Number of changed cells	300	□	190	3 h	26	S	[126]
Human erithrocytes	Erythrocyte osmotic fragility	Haemoglobin concentration	61	□	0.5	1 h	6	MC	[127]
Wistar rats	EEG spectral density	Alpha-rhythm power	170	⊙^∼^	50	21 d	?	MS	[128]
BALB/C mice	Whole blood characteristics	Blood leucocyte quantity	49	□	300	7 d	160		[129]
Cancer and endothelial cells	Hydrogen peroxide production	Peroxidase-induced fluorescence	19	□	2000	24 h	6		[130]
Soybean seeds	Gravitropism and germination	Gravitropism angle	26	□	110	24 h	15	S	[131]
Land snail Helix albescens	Nociception parameters	Latency of response to heat	15	□	10000	6 d	200	MCS	[132]
Rat satellite skeletal muscle cells	Membrane receptor activity	Intracellular Ca^2+^ level	42	□	300	1 h	?	MCS	[133]
Frog Xenopus laevis	Abnormal morphogenesis	Percentage of malformed embryos	190	□	105	4 d	200	S	[134]
Drosophila melanogaster	Ability to survive ionizing radiation	Survival percentage	61	□	1700	365 d	14		[135]
Solutions of substances	alpha-tocopherol properties	Size of nanoassociates	130	□	10	18 h	?	MS	[136]
Solutions of substances	Physico-chemical properties	Presence/absence	100	□	10	18 h	?	MS	[137]
BALB/C mice	Behavioral characteristics	Horizontal activity	25	□	20	1 d	?	MC	[138]
Arabidopsis thaliana	Gene expression	PHYB expression	38	⊙	50	10 d	36		[139]
Wistar rats	Behavioral patterns	Rate of interindividual interactions	140	⊙^∼^	50	25 d	75		[140]
Human spermatozoa	Cell motility	Velocity	110	⊙	150	20 h	?	CS	[141]
Micromycete U. consortiale	Anomalous mycelium growth	Pattern formation	100	□	100	7 d	45	MCS	[142]
Micromycete Neurospora crassa	Anomalous mycelium growth	Pattern formation	100	□⊙	10	7 d	45	MCS	[143]
Wistar rats	Aggressive interactions	Rate of the interactions	1100	⊙^∼^	50	21 d	75		[144]
Wistar rats	Gene expression	Number of c-fos producing cells	97	⊙^∼^	50	21 d	75		[144]
Outbred rats	Level of depression	Depression index	65	□	10000	160 h	200	MCS	[145]
Species of Daphnia	Lifelong development	Brood size	74	⊙^∼^	15	60 d	25	M	[146]
Human neuroblastoma cells	Proliferation rate in G1-phase	Absorbance 450 nm	45	□	200	48 h	27		[45]
Wistar rat lymphocytes	ROS level	Rate of fluorescence	25	⊙	5	2 h	17	MS	[147]
Arabidopsis thaliana	Growth rate	Biomass accumulation	37	⊙	1300	35 d	36		[148]
Wistar rat brain	Sensitivity to weak 42.3 GHz EMF	Life-span of epileptic foci	37	□	50	3 h	?	M	[149]
Wistar rats	Weight and hemodynamics	Weight	50	□	1200	1 w	60		[150]
Solutions of substances	Physico-chemical properties	Electrical conductivity	54	□	10	24 h	?	MS	[151]
Mice	Nociception parameters	Latency of response to heat	19	□	10000	200 h	200	MC	[152]
Japanese quail	Developmental abnormalities	Number of abnormalities	100	⊙	630	10 d	26	MS	[153]
Sprague-Dawley rats	Blood serum trace elements	Iron concentration	45	□	300	28 d	150		[154]
Cyanobacteria Synechocystis	Fluorescence spectra	Ratio of spectral peaks	8.5	□⊙	230	24 h	?	MCS	[155]
Human neuroblastoma cells	De-regulation of a few thousand genes	mRNA expression of 17 genes	150	□	200	48 h	27		[156]
C57BL/6 mice	Blood routine analysis	Neutrophil level	30	⊙	550	30 d	35		[157]
Snail P. corneus	Embryonic and juvenile development	Teratogenic abnormalities	49	□	600	45 d	30	MCS	[26]
Planthopper	Characteristics of the development	Vitellogenin transcript level	93	⊙	500	1 d	60		[158]
Planarian Girardia tigrina	Animal morphology	Regeneration index	430	□	50*	30 m	?	MCS	[159]
Micromycete U. consortiale	Melanin production	Spectral analysis	100	□	100	14 d	?	MCS	[160]
Planarian Dugesia tigrina	Intensity of fission	Number of divided animals	39	□	100	4 h	?	MS	[161]
Arabidopsis thaliana	Gene expression	Chlorophyll transcription	940	□	800*	120 h	25	S	[162]
Arabidopsis thaliana	Flowering	Delay in flowering time	16	⊙	1300	35 d	36		[163]
C57BL/6 mice	Blood properties	Serum noradrenaline level	12	⊙	500	30 d	35		[164]
Crucian carp	Enzyme activity in the intestine	Proteolytic activity of mucosa	43	⊙	15	1 h	25	MS	[165]
Planthopper macropterous females	Characteristics of the development	Gene Cry expression	68	⊙	500	12 d	60	M	[166]
Human neuroblastoma cells	Cell properties	F-actin density	19	⊙	470	48 h	35		[34]
Outbred rats	Level of aggression	Mean 5-level score	270	□	10000	190 h	200	MC	[167]
Human	Blood microcirculation	Capillary blood velocity	17**	⊙	200	1 h	120	MS	[168]
Mouse skeletal muscle cells	Cell viability	ATP content	44	⊙	2300	3 d	20		[169]
Human VH-10 fibroblasts	Content of DNA double-strand breaks markers	Number of cells with *γ*-H2AX foci	540	□	200	2 h	26		[170]
Seeds Lactuca sativa	Rate of germination	Number of sprouted seeds	20	□	20*	25 h	30		[171]
Crucian carp	Calpain activity	Optical absorption	74	⊙	500	1 h	25	M	[172]
C57BL/6 mice stem cells	Proliferation of cells	Number of large neurospheres	950	□	550	7 d	100		[169]
A. thaliana wild/cry-mutant	Gene expression	Gibberellin level	52	⊙	1300	33 d	36		[173]

Type of exposure: □—shielding, ⊙—compensation. Size of exposure system is in cm. HMF is in nT. Relative effect **E** is in %. *—there is *H*-dependence of the magnetic biological effect, **—statistically insignificant effect, ^∼^—servo control. Question column indicates that there are questions to description of M—the system of magnetic exposure, C—control measurements, S—statistical analysis.

#### Data extraction

On obtaining the full-text articles selected, one of us (VNB) analyzed and extracted their data to collect them in a table. This was made as follows.

We were particularly interested in the magnitude of the observed effects. If there were any common biological patterns—common to all organisms at all—they would have to manifest themselves in the form of a correlation between the magnitude of the effects and the physical factors of magnetic exposure. Consequently, our first goal was to extract data on these quantities.

Due to the variability in the reported essential parameters, however, the extraction of data from the articles caused some difficulties. In particular, many reports fall short in providing a complete description of either magnetic exposure or control conditions or statistical treatment used. In addition, different observables were reported in the same paper to change in HMF with various relative magnitudes. For this reason, it was difficult to make a completely accurate comparison of the MF effect values among different studies. To address these issues we have introduced an index of the magnetic effect magnitude that is applicable in a wide range of cases.

If *v*_0_ and *v* are sample means of the measured biological/biochemical parameter in control and under the exposure to HMF, respectively, the relative magnitude *E* of the effect may be defined in a number of different ways. These definitions have both advantages and disadvantages. For example, the definition *E* ≡ (*v* − *v*_0_)/(*v* + *v*_0_) is not suitable when a measured value changes its sign, and the simple definition (*v* − *v*_0_)/*v*_0_ does not make sense in the case of *v*_0_ ≈ 0. In these cases, it would be more correct to compare the difference between the means with the uncertainty of the means themselves. We need an objective definition that makes sense in all cases and is suitable for comparison of the effects in all situations. For this reason, the values of HMF effects that have been extracted from the articles are normalized in a certain way. They are derived from the original data in a standardized manner, as a positive index of the effect magnitude:

$$E=100\%\;{\left[\frac{{\left(v-v_{0}\right)}^{2}}{v_{0}^{2}+s^{2}+s_{0}^{2}}\right]}^{1/2}$$ (2)

where *s* is the standard error of the mean, or SE. This index generates reasonable values both when *v*_0_ = 0 and when *v* = −*v*_0_, and therefore is universally applicable to any measuring situation.

If the original paper lacked information on one of the two standard errors, we assumed them to be equal. If there was no information about SE, we recorded *s* = 0.1*v* and *s*_0_ = 0.1*v*_0_ (the mean value of SE/*v*, about 0.1, has been obtained from those of the selected articles, where *s* values were available). If the article did not informed on the type of variations—standard error or standard deviation—we assumed them SE. If for deviations, SD was provided as well as sample size *n*, then we converted SD into $\text{SE}=\text{SD}/\sqrt{n}$, otherwise we assumed *s* = 0.1*v*. Effects that have been described qualitatively in terms of “yes/no” were taken to be 100%. Parameters *v* and *s* that are necessary to calculate magnetic effect *E* according Eq (2) are available as supporting information “S1 Table”.

The endpoints observable in a study—if many have been traced—change under HMF exposure with different magnitudes that, in addition, depend on time. For this reason, we selected the maximal effect magnitude from each publication.

Extraction of other parameters, such as HMF values and times, was not difficult, although in a few cases the HMF value was estimated based on the accuracy of reported measurements.

### Results

The results (sorted by year from oldest to newest) are summarized in Table 1. For each reference, the Table shows the biological test system, taxonomic group, measured characteristic, the relative magnitude of the HMF effect, the type and size of the exposure system, HMF values inside and MF outside the system, the duration of exposure, and bibliographic source. Table 1 references 137 publications, which reflect an unbiased reporting of research results in this area.

The reported effects in Table 1 are almost all statistically significant at *p* < 0.05 or less as noted in each reference. In a few cases, the effects were not statistically significant, which is indicated with a double asterix superscript.

As said above, only the maximal magnetic effect was taken into account, if a few effects have been reported in the same article. It is also possible that studies in which the HMF effect was not observed may not have been published as it may be more difficult to publish null effects. Given these confounders, the ability to analyze this Table is limited. Nevertheless, a correlation analysis of the data was reasonable, and we were able to derive some generalizations.

**Fig 1:**
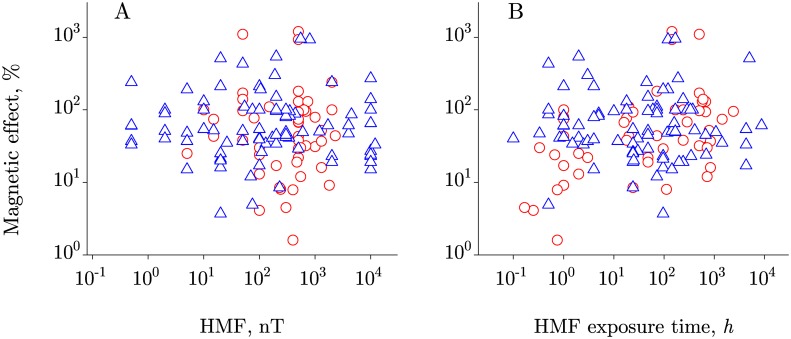
Correlation diagrams. Diagrams that show no correlation between the magnitude of a magnetic effect and the HMF value—A, or the duration of the HMF exposure—B, in groups Co (red) and Sh (blue). Pearson’s coefficients are in all cases less than 0.1 in absolute value, being negative, −0.07, for diagram A.

Fig 1 shows two correlation diagrams, where each point represents a study in which the relative effect *E* has been observed after exposure to HMF of a given value for a given time. The studies are divided into two groups, depending on the manner in which HMF has been obtained—by compensation (Co, red points) of the geomagnetic field, and by its shielding (Sh, blue triangles).

As can be seen, the relative magnitude of the effects is in the range of 1–1,000%, mainly 10–200%. There is no statistically significant correlation of the effect size with the HMF value or the exposure time. The effect size also does not correlate with their product, or “dose,” and with the size of exposure system that usually tells one about MF heterogeneity. This suggests that HMF and the duration of exposure can influence on the measured value only on par with many other hidden physiological factors, the effect of which should be considered mostly random in the set of various organisms.

**Fig 2:**
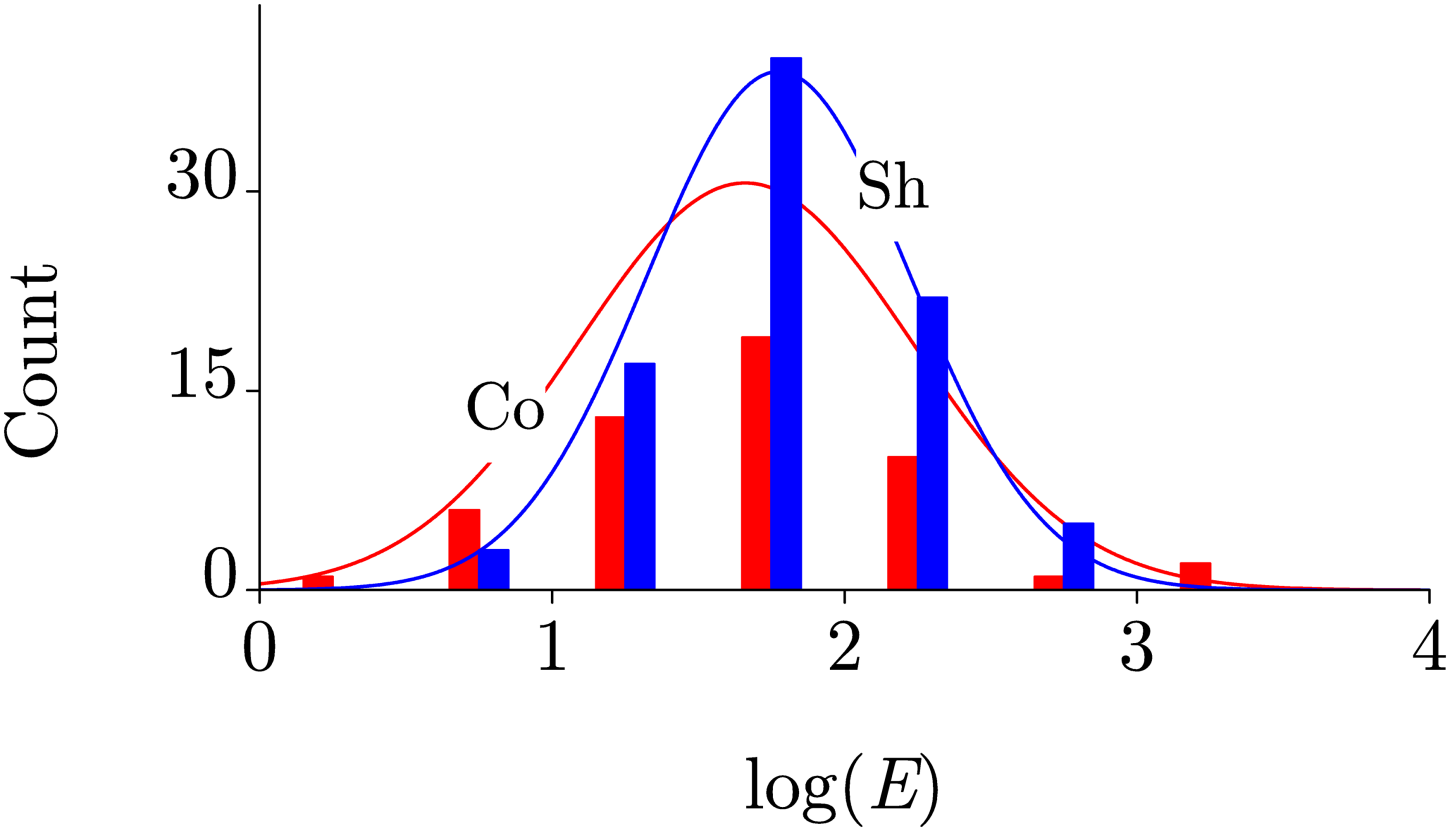
Distributions of the magnitudes of HMF effects. Distributions in groups of HMF obtained by compensating and shielding.

Distributions of the HMF effect magnitudes in groups Co and Sh are shown in Fig 2. They are close to log-normal one, and their means are not differ significantly. However, their variances are distinct at *p* = 0.03. Perhaps, this is the consequence of a closer similarity between shielding devices than compensating Helmholtz systems.

Other physical factors—temperature, pressure, optical and ionizing radiation, many chemicals—may have approximately the same range of effect magnitudes as that for MF effects. However, such effects are all more specific—they appear about the same way in different organisms. The absence of specificity in HMF biological effects is clearly seen in Fig 1, and this provides additional information about the nature of these effects. We suggest that the fact of no specificity is in a good agreement with our conjecture that there is no biophysical MF target common for all organisms. Only a physical target can be a general one.

Each work reviewed is unique and, therefore, not replicated by an independent scientific group. Rare exclusion is HMF nociception studies reported in [71, 81, 92, 97] and [112, 114, 132, 152]. Non-reproducibility is a common problem in laboratory magnetobiology. At the same time, this is also a fact that deserves attention. It agrees well with the statement that many physiological factors with their occasional contributions are involved in the formation of the HMF effect. This makes the replication an accidental coincidence rather than a regularity.

As over 95% of the studies referenced in Table 1 report significant effects of HMF, this biological effect is very well established but the mechanism remains unknown. Against the background of the diversity of observed magnetic effects, the primary MF target has not yet been identified. However, some generalizations can still be attempted. According to the data of the Table, closest to the primary physical level are studies that report on the involvement of gene expression in magnetoreception, e.g. [80, 108, 120, 123] and [139, 144, 156, 162, 166, 173], with different genes expressing in different situations. This suggests that the MF targets are rather physical and distributed, but they operate as magnetic receptors only in some specific conditions. Interestingly, it has been shown in [174] that adding/removing small segments containing a specific sequence into two different promoters switched the ability of a weak EMF to induce their gene expression.

A wide range of exposure times required for the appearance of the magnetic effect—from minutes to months—is another fact, indicating that there is no a single type of specialized biomolecules responsible for magnetoreception. Different biomolecules with very different characteristic speeds of functioning may become MF targets—the mediators of a magnetic signal transduction from the objects of the physical level to downstream systems and reactions.

As follows from the “Questions” column, there arise many methodological questions that are explained in a complete version of the Table available as supporting information “S1 Table”. Most important methodological problems are addressed in a special subsection “Methodological comments” in Discussion.

The conclusion drawn on the basis of Fig 1, that there cannot be a biophysical magnetoreceptor common to all organisms, is not completely obvious, and is a supposition rather than a logical inference. Since this supposition seems, however, to be significant both scientifically and methodologically, we present below details of this reasoning.

For this analysis lets simplify the equation for *E*. In Eq (2) lets neglect the contribution associated with the standard errors *s* that was shown do not exceed 0.1*v* in most cases. Recall that, with the exception of a few cases, the values of *E*_*i*_, *i* = 1, 2, … 137, represent statistically significant magnetic effects, and standard errors *s*_*i*_ refer to the corresponding mean values *v*_*i*_. If we express not in percentages and we omit the index *i*, then *E* ≈ |*v* − *v*_0_|/*v*_0_. Now suppose that a biophysical magnetoreceptor common to all organisms exists, and its response to MF at the level of MF-induced biophysical events is given by *H*-dependence in the form *f*(*H*), where *f*(*H* ≳ *H*_g_) ≈ 0. Since the observed magnetic effect *v* − *v*_0_ is linked to the primary biophysical magnetic response by a chain of low-predictable events at the biochemical and the following levels in different species, the linear statistical model of the observed magnetic effect is the following equation

$$v-v_{0}∼af\left(H\right)$$ (3)

where *a* is a random variable that maps the scaling of biophysical events to the observed biological level, and is distributed, due to a variety of concomitant factors, normally with zero mean. Then *E* ∼ (*a*/*v*_0_)*f*(*H*), and the covariance of *E* and *H* can be written, in a rough approximation, as follows

$$\text{cov}\left(E,H\right)∼\frac{\bar{\left|a\right|}}{\bar{v_{0}}}\int_{0}^{\infty }\;(H-\bar{H})\;[f\left(H\right)-\bar{f(H)}]\;\rho \left(H\right)dH$$

where *ρ*(*H*) is the idealized density distribution of the magnetic fields used in the experiments, and the line above the symbols denotes the mean value. For simplicity, suppose that the HMF effect is described by *f*(*H*, *H*_th_) = 0 for *H* > *H*_th_ and *f*(*H*, *H*_th_) = 1 for *H* ≤ *H*_th_, where *H*_th_ is a threshold level of MF, cf. Figs of sections “Universal physical mechanism” and “Intraprotein rotations.” Then the covariance takes a simple form

$$\text{cov}\left(E,H\right)\propto \int_{0}^{H_{\text{th}}}\;(H-\bar{H})\;\rho \left(H\right)dH$$

Hence it is clear that both for *H*_th_ → 0 and for *H*_th_ → ∞, covariance tends to zero, i.e., there is no statistical relationship between *E* and *H*. However, in the interval 0 < *H*_th_ < ∞, the covariance takes negative values, which could be observed in the experiment.

To show how this works in the case of quantities close to the experimentally observed, we performed a numerical simulation of the results presented in Table 1.

**Fig 3:**
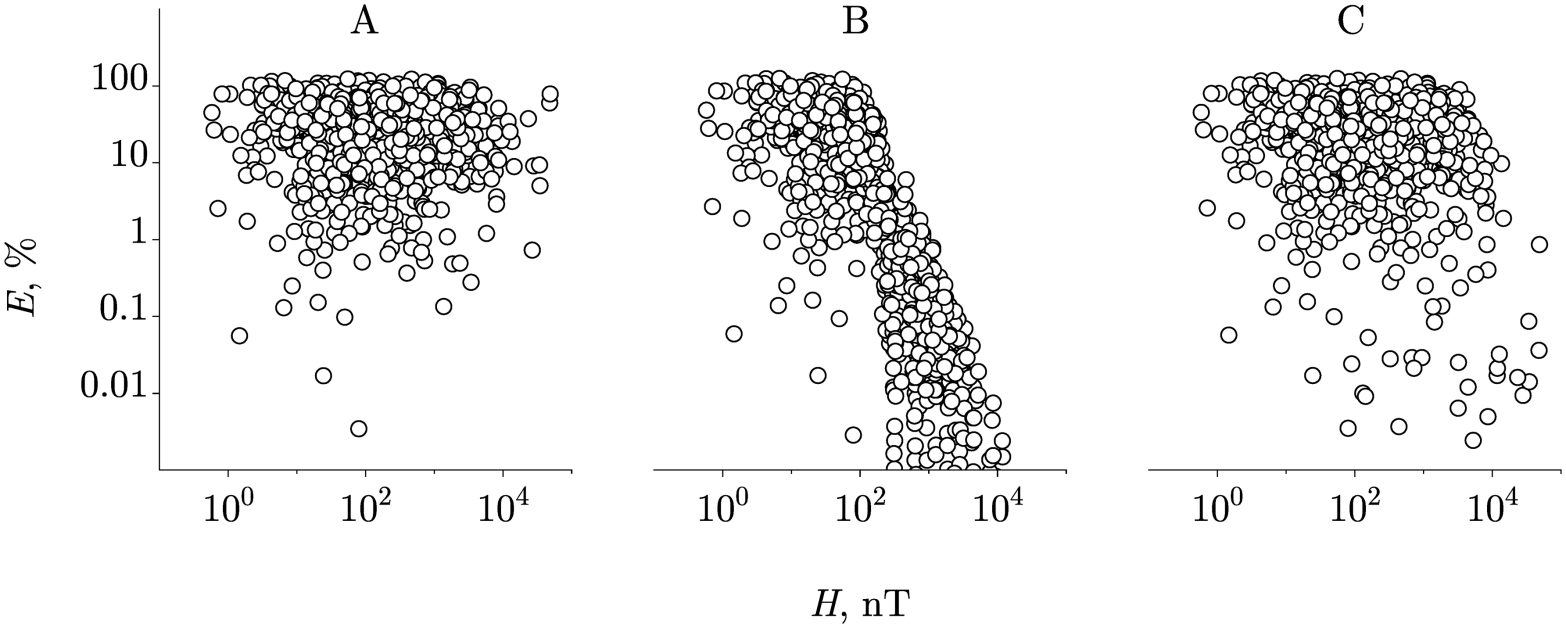
A numerical simulation of HMF effects. Effects that are expected in 1000 different biological species under the following model assumptions: A—unconditional HMF effects at *f* ≡ 1, B—HMF effects at *f* = *f*(*H*, *H*_th_) with a fixed *H*_th_ = 100 nT, C—HMF effects at *f* = *f*(*H*, *H*_th_) with a widely distributed *H*_th_.

First, it was established that the arrays of MF *H* values used in the experiments and of the measured control values *v*_0_ have approximately a lognormal distribution logN(*μ*, *σ*) with parameters *μ* = 5, *σ* = 2 and *μ* = 0.2, *σ* = 0.7, respectively. Using these distributions, sets of corresponding quantities were generated. To simulate the array of the effect values, Eq (2) has been applied, in which *v* − *v*_0_ was replaced by *af*(*H*, *H*_th_) according to the Model (3). The standard deviation of the normal distribution for the amplitude *a* of magnetic effects, *σ* = 1, was selected so that the array of the effects generated for the case *f*(*H*, *H*_th_) = 1 formed an average value and a deviation the same as in Fig 1A. The result of 1000 simulations is shown in Fig 3A. Since here we have put *f* ≡ 1, the effects in this case are considered only for selecting a correct amplitude distribution of *a* and cannot be used for conclusions about the character of the functional dependence *f*(*H*, *H*_th_).

Recall that our assumption was that there is one and the same biophysical MF target for all organisms. This means that there is one and the same function *f*(*H*, *H*_th_) with the same parameter *H*_th_ for all organisms. We have chosen *H*_th_ = 100 nT—the middle of the logarithmic interval of the fields in the experiments, used *v* − *v*_0_ = *af*(*H*, *H*_th_) and again performed a thousand simulations, Fig 3B. As is seen, there is a negative covariance, which corresponds to the above analytical reasoning.

An alternative hypothesis is that the MF targets in organisms are different and form largely a random set. This means, in terms of the above analysis, a significant difference in the threshold field *H*_th_ in different organisms. A wide distribution of *H*_th_ means the appearance of a significant subset, for which the covariance tends to zero. In other words, the expected picture is a significant decrease in the statistical relationship between *E* and *H*. For simulated values of *H*_th_, we used the absolute values of the realizations of a normal, log-widely distributed random variable with N(0, 10^4^). In this case, the correlation diagram *E*(*H*) had the form shown in Fig 3C. Apparently, indeed, there is a significant decrease in the statistical relationship of the magnetic effects with the magnitude of the MFs used.

Comparing Fig 3B and 3C with Fig 1A, one can conclude that the alternative hypothesis of a wide distribution of the threshold fields is more in line with the empirically observed pattern. What is the physical meaning of the wide distribution of threshold fields? As shown in this article, the equality, in the order of magnitude, *γHτ* ∼ 1 controls the appearance of the HMF effects. Consequently, the threshold value is the combination of the gyromagnetic ratio and the thermal relaxation time: *H*_th_ ∼ 1/γτ. A wide distribution of *H*_th_ means that at least one of the two values, *γ* or *τ*, does not have a fixed value in different organisms. In other words, either the magnetic moments themselves, i.e., primary MF *physical* targets, are different, or very different are local conditions that affect their relaxation. In both cases, this means that the *biophysical* targets are different, and therefore there is no biophysical MF target common for all organisms.

This analysis, of course, does not prove the assumption of nonexistence of a general biophysical target. However, this assumption does not contradict the experimental data of Fig 1A and, as one can see, has a rational justification.

We note that the simulated *E*(*H*) pattern with negative covariance is quite stable to the variation of model parameters; the traces of negative covariance are observed even in the case of logarithmically wide distribution of threshold fields *H*_th_, Fig 3C. This indicates the existence of a large number of targets, not only different, but also not obeying the statistical Model (3). Consideration of such targets will be carried out in a separate study.

From the viewpoint of theoretical physics, nothing but the very existence of the magnetic vacuum effect can be drawn from the current experiments, until sufficiently detailed MF-dependences of the effect are measured. Information on the nature of MF targets can be extracted from the MF-dependences only on the basis of comparison of experimental data and a theory. Experimental methods for the identification of the physical MF targets in the body require the parallel development of the theory of magnetoreception in terms of probability, physical properties of the primary MF target, and MF parameters.

Below, we will present several mechanisms of magnetoreception that are known from literature and often discussed with respect to specific and nonspecific magnetic biological effects. We will study their applicability in the limit of small dc MFs and show that not all of these are equally suitable for the explanation of the nonspecific biological effects of HMF.

Among those mechanisms of magnetoreception, there is one that is valid for both the ac MFs and HMFs. The mechanism does not depend on the characteristics of intermediate biophysical/biochemical stage of magnetoreception, and therefore is universally applicable to any magnetic targets of molecular nature. This fact makes it reasonable to attempt experimental verification of this mechanism.

## Theoretical concepts

In this section, we briefly survey the various mechanisms of magnetoreception and determine their predictive power with regard to the HMF biological effect. We have restricted the discussion to the theoretical mechanisms that are provided with sufficiently developed mathematical models and, as we have assessed, have the potential to explain the observation of effects in a HMF.

Some mechanisms, such as the ion cyclotron and parametric resonances, that were often discussed in the past, will not be included since they have never been able to overcome the thermal noise objection, e.g. [18, 175] p. 180–181. Also we do not consider the mechanism of electromagnetic induction, because our task is to identify those mechanisms that are general enough to describe effects of both the ac and dc MFs, including HMF mode.

Highlights in magnetobiology are well known: 1) the biological effect often occurs in EMFs that have only vanishingly small inductive or thermal action. Where it concerns nonspecific biological effects, 2) they can be strongly non-linear and, in particular, contain “windows,” where the effect decreases with growing MF [176], and 3) they are little with respect to predictability and reproducibility. The first fact, otherwise known as “*kT* problem,” is most surprising. It can be expressed by inequality *mH* ≪ *kT*, where *H* is the MF magnitude, *m* is the magnetic moment of the putative MF target, *k* is the Boltzmann constant, and *T* is the effective temperature of the target. For example, the magnetic energy of an electron in the geomagnetic field is 2.9 × 10^−9^ eV, which is seven orders of magnitude less than *kT* at physiological temperatures.

It is not clear how a change in magnetic energy that is much smaller than the scale of the thermal fluctuations, influences the rate of a chemical reaction, whilst this influence is necessary to excite a biological response. One could say that a correct explanation of magnetoreception should transform the above inequality to at least an approximate equality.

One of the main magnetoreception hypotheses argues that the MF target has a large magnetic moment. For example, magnetic nanoparticles in an organism may occur naturally as well as get into it from the outside, and this eliminates the problem [31]. However, non-thermal effects exist also in those organisms where no magnetic nanoparticles are known. Therefore, the quest continues for a molecular mechanism of magnetoreception. Often considered are the simplest microscopic single-particle or a few-particle systems: a charged oscillator or a rotator, and the spin magnetic moments.

With respect to the molecular mechanisms, and in order to overcome the above-mentioned inequality, one has to assume that the effective temperature of the target is small. This is only possible if dissipation effects that are caused by the interaction of a dynamic system with the thermostat are small. The dissipation can be neglected if the evolution of relevant degrees of freedom is completed before the thermal equilibrium is reached—i.e., the lifetime of those degrees should be less than their thermal relaxation time. Such degrees of freedom are known—it is for example, the intermediate spin-correlated states of a radical pair (RP) in spin-chemical reactions.

However, dissipation is not the only reason that prevents the MF signal to be transformed into the change in the rate of a chemical reaction. Another obstacle is inertia. The final change in the velocity of a generalized coordinate does not occur simultaneously with the application of the force, but shows a linear dependence on time. Accordingly, the energy and coordinate vary proportional to *t*^2^. Then, for small magnetic forces that are common to magnetobiology, one can redily prove the following statement: A particle with elementary charge and mass could gain the energy of the order of *kT* for a time which is just too long to be considered even in the absence of dissipation and at the most favorable interaction mode.

Are there inertia-free mechanisms? Yes—they are based on the laws of angular momentum and quantum phase dynamics. In this case, a final angular velocity of free precession occurs simultaneously with the application of a torque, and it does not depend on time explicitly. This is a consequence of the degeneracy of the rotational energy in the direction of the momentum. Thus, the direction of angular momentum or spin can be changed proportional to *t*, i.e., without inertia. A similar pattern holds for the quantum phase that is also not connected to particle energy.

Inertia-free are the most promising and often-discussed hypothetical molecular mechanisms of magnetoreception. They consider the influence of MF on (i) the rate of reactions that involve spin-correlated radical pairs [177, 178], (ii) quantum rotations of molecular groups inside proteins [179, 180]. Inertia-free are also mechanisms based on (iii) the local spin and structural ordering in liquid water [181]. A detailed analysis of these quantum mechanisms related to the Zeeman effect, as well as of many others, is presented in [18, 182].

Thus, there are simple physical arguments that, on the one hand, limit the list of possible magnetoreception mechanisms. Within this list, on the other hand, those arguments do not distinguish any one of the mechanisms, since no one has yet been identified experimentally, and they are all hypotheses.

Given these considerations, the mechanisms we have evaluated are: radical pair, universal phyisical, molecular rotations, target rotations, stable magnetic nanoparticles, superparamagnetic nanoparticles, and water protons.

### Radical pair mechanism

Some chemical reactions are known to change their rate in the MF due to formation of the intermediary magnetically sensitive state—a spin-correlated pair of radicals, e.g. [183, 184]. This is one of the most actively discussed magnetoreception mechanisms that is called Radical Pair Mechanism (RPM). The total spin of the radicals or the relative orientation of the spins affects the probability of the product formation. For example, probability of recombination of the newly formed radicals in a homolytic reaction depends on the relative orientation of their spins. A constant MF changes the likelihood of a favorable orientation and, thereby, able to shift the chemical balance. This mechanism does not possess a frequency selectivity in the low-frequency range, since the evolution of the magnetosensitive spin state occurs within a very short lifetime of the pair usually of the order of 10^−9^–10^−7^ s.

Spin-chemical magnetoreception has certain difficulties. Known effects in spin chemistry appear in a relatively strong MF, and their possible involvement in magnetoreception is not yet established for sure. Maximum values of only about 0.1% are estimated for the effects of MFs like the geomagnetic field, even in the favorable case of large thermal relaxation time of the electron spin states [185]. Finally, there is no laboratory example where an MF less than the geomagnetic field would significantly change the rate of a biradical biochemical reaction: one cannot trace the result of the action of an MF of the order of units of *μ*T on such a reaction in vitro.

It is very likely, however, that RPM actually works in the case of specific magnetoreception in migratory animals. It has long been conjectured that photochemical reactions that generate spin-correlated biradicals underlie a compass sense in migrant species [186]. This idea is in good agreement with the fact that the eyes of birds often mediate their magnetic reactions. It was found that formation of biradicals takes place in the cryptochrome molecules in the retina of the bird’s eye. Since there is an orderly arrangement of the immobile photo-receptors with cryptochromes in the retina, they form an oriented array of *N* elements that can provide an enhanced sensitivity. The sensitivity of the visual system as a whole to MF changes may increase in proportion to $\sqrt{N}$. The fact that magnetic susceptibility in some birds depends on the spectral range of the optical radiation and on its intensity supports this hypothesis. The fact that a hypothetical RPM-based magnetic sensor reacts equally to the oppositely directed MFs is also in accord with a significant part of observations in animal magnetic navigation.

With regard to non-specific magnetoreception, RPM is certainly possible, but unlikely due to (i) the lack of frequency selectivity, (ii) small magnitudes of the effect, and (iii) lack of the laboratory examples of its fitness. Experiments [187] regarding the MF influence on the growth and gene expression in *Arabidopsis thaliana* in MFs of 0.05–100 mT had to prove the involvement of cryptochromes in magnetoreception in plants; however, one has failed to confirm these results [188]. In [162], it is suggested that the RPM cannot while the quantum interference mechanism [189] can explain weak MF-dependences of gene expression in plants that are observed in this study. These failures of the RPM are probably due to irregular arrangement of the cryptochrome flavoproteins in plants, as opposed to the bird’s eye. Perhaps, the biradical mechanism is just one of the necessary elements of magnetoreception.

For example, a combined mechanism that considers biradical reactions in the presence of magnetic nanoparticles eliminates the difficulties of nonspecific biradical magnetoreception. Magnetic nanoparticles have a magnetic moment, and therefore they are sources of the endogenous MF. This field is nonuniform and rather strong, of the order of 1–100 mT near the nanoparticles [31]. In contrast to weak external MFs of the geomagnetic level, this strong MF can lead to a significant shift in the rate of biradical reactions by the suggested mechanism of S-T mixing in strongly inhomogeneous MF [31], while the nanoparticles themselves are rotated under the weak external MFs. This concept has been further developed in [32, 190, 191].

Can biradical mechanism explain the effect of a zero-MF? Apparently, no. Judging by the experimental MF-dependences of the biradical reactions rate, which are available in the original literature, there are no peculiarities in the MF close to zero, see references in [192]. In the fields that are less than 0.1 mT, the dependences are very smooth, which means that the zero-MF effect as defined above is absent. This is not by chance.

The natural state of a biradical is a superposition of the singlet S (↑↓) and triplet T (↑↑) states. Leaving aside many details, one can say that the quantum levels of these states are separated by an interval of the order of electron energy in MF *H*, i.e., Δ*ε* = *μ*_B_*H*, where *μ*_B_ is the Bohr magneton. The magnetic effect is associated with transitions in these states. There are general restrictions on observation of S-T transitions and, therefore, on the magnetic effects in biradical reactions.

One of the results of quantum mechanics is the following statement. It is impossible to measure the change in energy Δ*ε* of a quantum system in a time less than Δ*t* ∼ ℏ/Δ*ε*, because the act of measuring itself makes the measured value less certain in the value of the order of ℏ/Δ*t* [193] p. 157–159. A biradical must keep coherence for at least Δ*t* in order to allow measuring its energy changes. In the biradical reactions, the decay of the coherent state of the pair to form a whole molecule or a pair of free radicals should be considered an act of measuring. Accordingly, the duration of measurement is the biradical lifetime that is limited by its thermal relaxation time *τ*, among many other factors. In other words, for observation of electronic chemical magnetic effects in weak MFs, it is necessary that inequality *τ* > *ħ*/*μ*_B_*H* be fulfilled, or *τ* > 100 ns for MFs of about the geomagnetic field. The existence of electronic states with so a large thermal relaxation time is questionable. There are other difficulties, which are mainly the insignificant magnitude of magnetic effects and the absence of a characteristic threshold MF value in the range of HMFs.

For many biradical reactions, characteristic is that the yield of free radicals increases with the MF growing from zero to the fields on the order of the hyperfine interaction, i.e., 1–100 mT. This is the so-called Low Field Effect (LFE) [192, 194]. Experimentally observed changes do not exceed a few percent. In [192], the canonical case has been studied of the S-T conversion in a pair of radicals, one of which had a magnetic core with spin 1/2 (a proton). The Zeeman energy of the electrons and the isotropic hyperfine interaction of one of the electrons to its nucleus were taken into account. Other interactions and spatial dynamics of the radicals were not considered. An approximate analytical solution for the LFE has been found:

$$E\left(H\right)\equiv E_{\text{lfe}}={\left[\frac{5}{2}+\frac{10}{{\left(\gamma_{e}H\tau_{e}\right)}^{2}}\right]}^{-1}$$ (4)

where *γ*_e_ is the electron gyromagnetic ratio, *τ*_e_ is the lifetime of the coherent state of electron pair. Let one assume that *τ*_e_ approaches the necessary value of *τ* of about 100 ns, even though several other physicochemical factors also limit the lifetime. Even with this incredible condition, the critical MF that corresponds to a half-maximum effect, as follows from Eq (4), is *H* ∼ 2/*γ*_e_*τ*_e_ ∼ 1 mT. It is very far from 1 *μ*T required by the HMF effect.

A recent review [23], after many preceding original publications, supports that RPM underlies the compass sense, i.e., a spin-chemical cryptochrome reaction in a bird eye depends on the MF direction with respect to the reactant molecules. Due to the anisotropic caracter of the hyperfine interaction responsible for that magnetic effect, the effect of changing the MF direction and that of the MF switching on/off are of the same order of magnitude. This means that the above restrictions are valid for this case also. Indeed, Fig 7 of that review presents relative values of the magnetic effect that have been numerically calculated at different coherent spin evolution times that, of course, are always not greater than the thermal relaxation time. The values for changing MF of 50 *μ*T have been 6.7% for *τ* = 5 *μ*s and 0.6% for 0.5 *μ*s. As can be estimated, for the ultimate value *τ* = 100 ns, the effect would be about 0.1% at 50 *μ*T and 0.002% at 1 *μ*T, which is at obvious variance with the HMF effect data.

To be fair, a few other theoretical models with sets of interactions which could be viewed as more realistic than that in [192] predict somewhat higher sensitivity. However, their results are also strongly dependent on time constants, the choice of which are not always indubitable. In the model [195] that describes a coupled chain of flavin–tryptophan biradicals, the relative reaction yield at the RP lifetime of the order of 100 ns has been numerically calculated to be about 5% in a MF of 100 *μ*T, however with almost linear *H*-dependence in this range of small MFs. This, of course, excludes the possibility of applying this model to the HMF effects. Moreover, the model is hardly applied where its implicit assumption is not valid, that the electron thermal relaxation time is greater than the chemical time constants. Even greater magnetic effect of about 18% per 100 *μ*T is calculated in [196] for a hypothetical cryptochrome RP of the flavin cofactor with the superoxide radical. However, again, the largest chemical time constant has been chosen to be as large as 1 *μ*s, while the thermal decoherence has not been addressed. It is stated that such long lifetimes are characteristic for biological systems, but no direct experimental evidence in vitro has been adduced in the form of a MF-dependence.

Experimentally observed MF sensitivity, e.g., in cryptochromes in vitro, is about 1% per mT, e.g. [197]. In this latter study, a chemical amplification of the magnetic effect in cryptochrom biradicals have been found. This nearly tenfold amplification is suggested to support a cryptochrome-based magnetic compass sensor in animal navigation. However, as is seen from the above estimate, this is still far from enough to explain nonspecific HMF effect.

It follows that RPM is of little use to explain nonspecific biological effects of zero MFs, although its involvement in the specific magnetic sense of birds and other species is plausible. This conclusion could be challenged if a mechanism of biological amplification of smallest primary MF-induced changes, other than the eye cryptochrome immobility and ordering, will be revealed in the future.

### Universal physical mechanism

In [36], it has been considered a nonuniform precession of magnetic moment under the action of the MF, whose direction is unchanged while the value changes. This MF does not cause quantum transitions; hence the classical model of the Larmor precession is sufficient. It turns out that this precession mode has interesting properties, similar to those of non-specific magnetoreception.

The precession of magnetic moments in MF precedes any biophysical or biochemical mechanism of magnetoreception, and largely determines the spectral and non-linear characteristics of the biological response. Objects in biological cells that possess a precessing magnetic moment are unpaired electrons, paramagnetic ions, protons and other magnetic nuclei. Protein-bound ions and the rotations of molecular groups with a distributed electric charge may also have a virtual magnetic moment.

The mechanism considered is based on the following points: the external MF acts on a magnetic moment; the magnetic moment precesses and undergoes thermal relaxation. A biological effect occurs if, during the relaxation time or faster, the MF imparts a significant disturbance to the dynamics of the magnetic moment. A measure of the disturbance is a deviation from the state of the unperturbed uniform precession in the GMF. Idealizations of the model: 1) uniform precession is a natural background for the microscopic events in organisms, 2) the events of the next level—biophysical or biochemical events—form a nonhomogeneous Poisson process, the rate of which varies periodically with the precession phase in the local coordinate system of the target, 3) biological effect is associated with the disturbance of precession of the magnetic moments of the same type—the disturbance being averaged over time and over the realizations of random precession phase. Of course, the biological effect is observed only when the biophysical-level changes go through the stages of transformation at the biochemical, physiological and biological levels of the system.

In this scenario, the average probability of biophysical events *P*(*H*, *h*, Ω, *γ*, *τ*, *β*) depends on six values. Three of these are MF variables: the constant MF component *H* and the amplitude *h* and frequency Ω of the variable component. Three others are MF target parameters: the gyromagnetic ratio *γ* and the parameters *τ* (relaxation time) and *β*, describing respectively the thermal and the “signal” interaction of the magnetic moment with its immediate environment.

The change in average probability under exposure to an ac/dc MF, i.e., Δ*P* ≡ *P*(*H*, *h*, …) − *P*(*H*, 0, …) has the following approximate form [36]:

$$\Delta P=\frac{1}{4}\beta^{2}\tau^{2}e^{-\beta \tau }\;[{\;\text{sinc}\;}^{2}\;(\gamma H\tau /2)-\sum_{n}J_{n}^{2}\;(\frac{\gamma h}{\Omega }){\;\text{sinc}\;}^{2}\frac{(\gamma H+n\Omega )\tau }{2}]$$ (5)

Here and below, in order to significantly simplify mathematical equations, the arguments of functions are either omitted or given in an abbreviated form—only those arguments are displayed that are relevant to the context of a given equation. At *h* = 0, under exposure to a dc MF that is decreasing from the geomagnetic field *H*_g_ to an HMF *H*, the probability change, i.e., Δ*P*_dc_ ≡ *P*(*H*, 0, …) − *P*(*H*_g_, 0, …), is equal to

$$E\left(H\right)\equiv \Delta P_{\text{dc}}=-\frac{1}{4}\beta^{2}\tau^{2}e^{-\beta \tau }{\;\text{sinc}\;}^{2}(\gamma H\tau /2)$$ (6)

where it is assumed that sinc^2^(γ*H*_g_τ) ∼ 0. This Eq (6) is for the HMF effect that we associated above with *E*(*H*).

It is seen that the effects of the ac MF at *H* = 0 depends on *γh*, while the HMF effect depends on *γHτ*. The difference can be used to extract information about the gyromagnetic factor and the thermal relaxation time separately from experimental MF-dependencies. This can be done as follows.

Consider two dependencies: 1) Δ*P*_dc_(*H*) and 2) Δ*P*(Ω) at *H* = 0 and at a certain value of *h* that will be given below.

**Fig 4:**
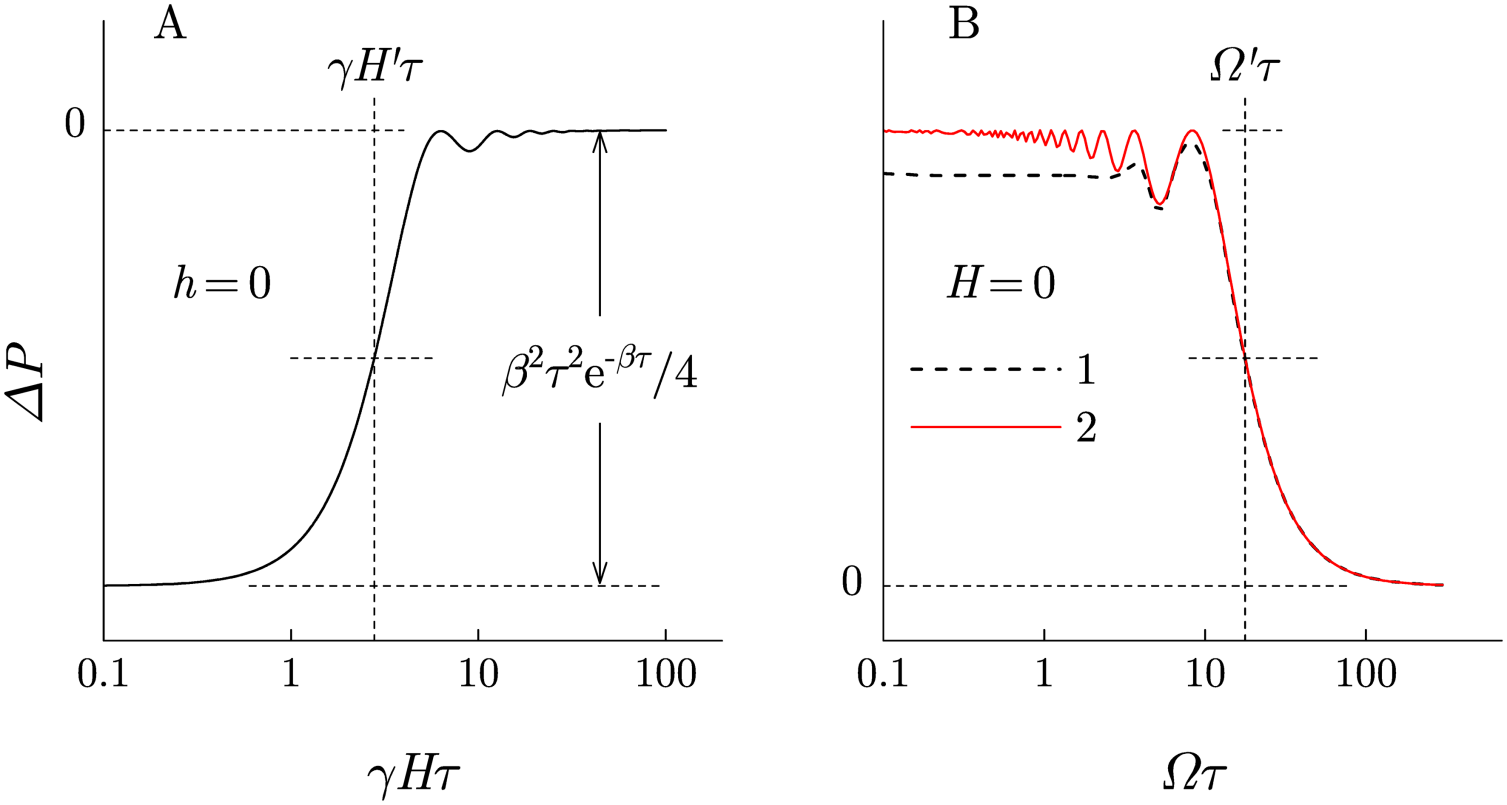
The change in the probability of primary reaction as a function of different variables. (A) The probability change with a decrease in the constant MF, i.e. the “zero-field” effect, Eq (6). (B) The probability change with a decrease in frequency of the alternating MF at *ηh* = 20, 1—according Eq (7) and 2—according Eq (8).

The first dependence is shown in Fig 4A. The argument *γHτ* ≈ 2.8, at which the curve reaches the middle between the initial and final levels, does not depend on *β*. Having found the corresponding value *H*′ from an experiment, one can define the product of the primary target parameters: *γτ* = 2.8/*H*′. Further, we assume that the value of *η* ≡ 2.8/*H*′ is known, so that *γ* = *η*/*τ*.

In the second case, at *H* = 0 and with account of the relation *γ* = *η*/*τ*, Eq (5)—that now can be conveniently denoted as Δ*P*_ac_—takes the following form:

$$\Delta P_{\text{ac}}=\frac{1}{4}\beta^{2}\tau^{2}e^{-\beta \tau }\;[1-\sum_{n}J_{n}^{2}\;(\frac{\eta h}{\Omega \tau }){\;\text{sinc}\;}^{2}\left[n\Omega \tau /2\right]]$$ (7)

For sufficiently large values of *ηh* = 2.8*h*/*H*′ > 10, Eq (7) can be simplified considerably. Note that because *H*′ is mostly less than 1 *μ*T, the “large” values of *h* for large *ηh* begin with a few *μ*T. Due to the properties of the Bessel functions, only those terms of the sum contribute significantly in Δ*P*, for which the argument of the Bessel functions is of the order of unity. That is, the values of *Ωτ* must also be as large as *ηh*. But then, by virtue of the properties of the sinc-function, only members of the sum with *n* = 0 make a major contribution. Hence, relation Eq (7) can be written as

$$\Delta P_{\text{ac}}=\frac{1}{4}\beta^{2}\tau^{2}e^{-\beta \tau }\;[1-J_{0}^{2}\;(\frac{\eta h}{\Omega \tau })]$$ (8)

Functions (7) and (8) that are shown in Fig 4B imply that the approximate Function (8) can be used to determine the value of the argument Ω′*τ* at which there is a strong change in Δ*P*_ac_. This value is determined by the position of the first extremum in the derivative of the function $J_{0}^{2}\left(x\right)$, i.e., *x* ≈ 1.1. Hence we find *ηh*/Ω′*τ* = 1.1 and, finally, using the definition *η* = *γτ* = 2.8/*H*′,

$$\tau \approx 3\frac{h}{H^{\prime }\Omega^{\prime }},\;\;\gamma \approx \frac{\Omega^{\prime }}{h}$$ (9)

where *h* is the amplitude of the ac MF used in the experiment. We rounded the numbers up to one significant figure: only the orders of magnitude make sense, as this theory relates with the experiment only through less predictable MF-signal transduction at the biophysical, biochemical, and higher levels.

Thus it is clear how to determine the gyromagnetic ratio and the thermal relaxation time of the precessing primary magnetic moment. First, on the basis of preliminary dc-experiments, one should set a level of the static MF *H*_min_, where the biological response is almost unchanged with further reduction of *H*. Second, it is necessary to obtain experimental dependences of the biological effect (i) *E*(*H*) at *h* < *H*_min_ in a dc-experiment, and (ii) *E*(Ω) at *H* < *H*_min_ and *h* > 10*H*_min_ in a ac/dc-experiment. Inflection points *H*′ and Ω′ of these dependences will identify the constants *γ* and *τ* according to relations Eq (9).

The above-described HMF effect in the dc MF and the effect in the ac MF are useful. If the HMF effect is due to the non-uniform precession of magnetic moments, then 1) the effect is about three times greater in magnitude [36] and much more likely than the frequency selective ac/dc magnetic effect, since magnetic moments of all types respond to the HMF, and thus those associated with a biological measurand will respond inevitably; 2) revealing of the HMF effect does not require selection of the MF frequency, see Fig 4A; 3) the measurements allow (for the first time) to determine two parameters of the precessing moments at once—their gyromagnetic ratio and thermal relaxation time.

**Fig 5:**
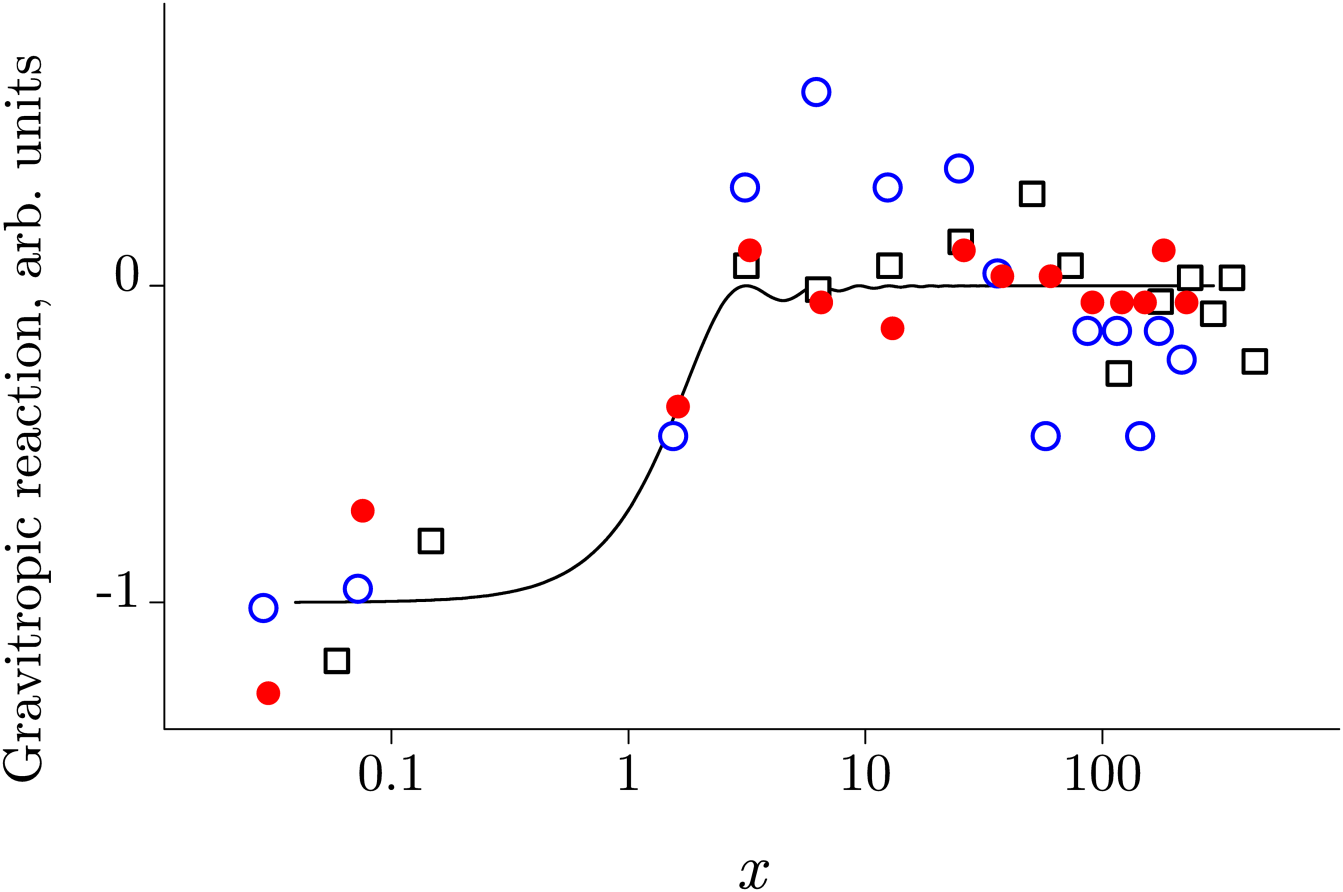
Approximation of curve −sinc^2^(*x*) with *x* = *γHτ*/2 by experimental data [96]. Designations: the number of roots growing horizontally ▫ or vertically •; growth angle of the remaining roots ○.

As an example, *H*-dependences of the gravitropic reaction in watercress roots [96] are shown in Fig 5. Experimental data of a few measured parameters were approximated by the Levenberg–Marquardt algorithm. An approximating function, in accordance with Eq (6), was the function *k* sinc^2^(*k*′*H*) + *k*″, where *k*, *k*′, and *k*^′′^—coefficients that minimize the standard deviation of experimental points from the curve. In other words, the fitting was achieved by scaling and offset of the squared sinc-function along the ordinate, and by the abscissa scaling. Then, the obtained curve, already together with the experimental points, was subjected to an inverse transform. Therefore, fitting functions of all the measured parameters were reduced to the same *H*-dependence, −sinc^2^(*x*) with *x* = *γHτ*/2. This is convenient for visualizing the overall of dependencies. Thus, the subjective factor was totally excluded from the comparison procedure of the experimental *H*-dependencies with a common motive −sinc^2^(*x*).

This physical mechanism predicts that deprivation of both dc and ac MFs is equally important for observing the magnetic vacuum effect. Removal of only one of these MFs would be insufficient. Experimentally, the interference of the HMF effects, at dc MF compensation, with the effects of geomagnetic storms has been observed in [91] and with the effects of the background EM noise in [136, 137, 198].

Interestingly, the universal mechanism predicts the absence of the primary effect in strong MFs at the level of Tesla. This is due to a 2*π*-rotational symmetry of the phase of magnetic moments precessing in their local molecular environment. The characteristic precession and magnetic resonance frequencies change proportionate to the MF value. As shown in [36], observation of the nonuniform precession effects (that are proportional to approximately $J_{1}^{2}(h/H)$ at Ω = *γH*) becomes more difficult in a strong constant MF, although observation of the resonance is facilitated. In a strong MF, the resonance can be observed at a lower ac MF amplitudes, but the local effects of the inhomogeneous precession are only possible at larger amplitudes. Therefore, in a strong constant MF, the possible effects of inhomogeneous precession simply disappear, which is well seen in Fig 4A. Perhaps this explains the relative safety of a *short-term* exposure of organisms to the MFs of the diagnostic procedure know as magnetic resonance imaging. However, other biological effects can develop due to the growing contribution of spin-correlated biradicals.

Finally, this primary physical mechanism, while predicting the maximum effect in ac/dc MF at *h* = 1.8*H* and Ω = *γH*, is in agreement with numerous experimental data, a review of which is available in e.g. [18] p. 307–314.

A particular characteristic of this mechanism is that it predicts the form of MF-dependencies for HMF and ac/dc MF effects in the same biological system. Therefore, its most effective validation would be to compare these dependencies. How to validate this theory is discussed in detail in [36]. We note that in terms of quantum mechanics, this mechanism is not connected with quantum transitions. In opposite, these are a hindrance to its implementation [199, 200], and therefore a significant deviation from parallelism of the fields **h** and **H** destroys the ac/dc MF effect.

### Intraprotein rotations: Molecular gyroscope mechanism

Interestingly, the magnetoreception mechanisms that are presented above involve, in one way or another, a variety of rotations: the precession of magnetic moments of different nature and the rotations of nanoparticles that have their own intrinsic magnetic moment. The presence of rotations in the models of magnetoreception is not by chance. This is a consequence of the specificity of the MF interaction with matter. This link is carried out only through the interaction with magnetic moments. The magnetic moments, on the one hand, experience a torque in the MF, and, on the other hand, are bound with spins or can be generated by rotations of electric charges.

The following briefly shows a mechanism [180, 201] developed from study [202]—the so-called molecular gyroscope—and derives its properties in the HMF.

The essence of the mechanism is a rotation of large fragments of macromolecules or amino acid residues with distributed electric charge. Due to this rotation, a magnetic moment appears that interacts with an external MF. In the quantum description, the rotation of fragments is an interference of their angular states with a nonzero magnetic quantum number.

How can the molecular gyroscopes arise? The folding of long protein chains that are synthesized by ribosomes results in the formation of protein globules. In the process of the folding/maturation, their occurs an evolutionary-determined and workable conformation of a protein molecule. Incorrect folding can break or make impossible a specific function of the protein. The characteristic time of folding is highly dependent on the length of the protein chain, ranging from microseconds to seconds [203, 204]. At some stages of the folding, virtual cavities free of water molecules may occur within the protein. Indeed, hydrophobic cavities of the order of 1 nm or less push out water molecules [205]. In these cavities, the amino acid residues (molecular gyroscopes) rotate within milliseconds whilst looking for the best position. That would be enough for the action of ELF MFs on such rotations and, therefore, on the path and the result of the protein folding.

The magnetoreception mechanism that utilizes the interference of quantum states of a molecular gyroscope is based on the fact that the main rotational degree of freedom of a gyroscope can long remain non-thermalized, or “cold.” Rotations of small molecules that are fixed inside virtual cavities are well protected from the thermal vibrations of the cavity walls and, therefore, have a relatively long lifetime. In combination with quantum interference effects, these coherent long-lived rotations allow one to explain magnetoreception. In this way the molecular gyroscope mechanism resolves the *kT* problem [206].

What does the gyroscopic mechanism predict regarding the zero-MF biological effects?

We would like to recall the following. The fact that the best position of an amino acid residue has been found in the process of folding is considered the result of some reaction. The reaction yield, or the number of gyroscopes having entered this reaction, or alternatively, the equilibrium number of gyroscopes is associated with a biological effect.

The general result of the gyroscopic model of magnetoreception is a relation for the time-averaged reaction probability *P* under exposure to a dc MF *H* and a collinear ac MF of the amplitude *h* and frequency Ω,

$$P≃p+\frac{w\tau }{2}\sum_{mm^{\prime }n}\sigma_{mm^{\prime }}^{2}\;{\left|\frac{\sinh(b\tau )}{b\tau }\right|}^{2}J_{n}^{2}\;[\left(m-m^{\prime }\right)\frac{\gamma h}{\Omega }]$$ (10)

where *p* is MF-independent part of the probability, *w* is the rate of new gyroscope creation events, *τ* is the thermal relaxation time, *m* or *m*′ is the magnetic quantum number, *σ*_*mm*′_ are the density matrix elements that describe the initial state of a gyroscope, *γ* is its gyromagnetic ratio, J*_n_* is the *n*-th order Bessel function of the first kind, and

$$b\tau \equiv 1+i\tau [\omega_{mm^{\prime }}-\left(m-m^{\prime }\right)\gamma H-n\Omega ]$$

In this notation, *ω*_*mm*′_ = ℏ(*m*^2^ − *m*^′2^)/2*I*, where *I* is the moment of inertia of the gyroscope. In the current work, we will study a special case of HMF, that is, *h* = 0. Note that if *m* ≠ *m*′ or *m* ≠ −*m*′ then *P* = *p*, because in this case *τω*_*mm*′_ ≫ 1 and the sinh group in Eq (10) turns to zero. Evidently, one should take only those terms of the sum in Eq (10) where *n* = 0 because *h* = 0, and *m*′ = ±*m*. After some algebra, we obtain the formula

$$P≃p+w\tau S,\;\;S\equiv \frac{k}{2}\sum_{m}\;\sigma_{mm}^{2}+\frac{1}{2}\sum_{m\neq 0}\;\sigma_{m,-m}^{2}\frac{k+\sin^{2}\left(2m\gamma H\tau \right)}{1+{\left(2m\gamma H\tau \right)}^{2}}$$ (11)

where *k* ≡ sinh^2^(1) ≈ 1.38.

A rotator in the form of an amino acid residue has a significant moment of inertia, in a quantum scale. For this reason, Boltzmann statistics is used to assess the populations of the gyroscope rotational states. The energies of the rotational states are *ε*_*m*_ = ℏ^2^ *m*^2^/2*I* − ℏ*γHm*. Since the Zeeman splitting ℏ*γH* in this case is much less (∼ 10^-6^) than the energy of rotational levels, this splitting can be ignored in the Boltzmann distribution. Thus, *σ*_*m*,±*m*_ = exp(−*αm*^2^)/*Z*, where *α* ≡ ℏ^2^/(2*IkT*) is a coefficient and *Z* = ∑_*m*_ exp(−*αm*^2^) is the statistical sum. Further, one will need a notation

$$a\equiv \frac{k}{2}\;\sum_{m}\;\sigma_{mm}^{2}=\frac{k}{2}\frac{\sum_{m}\exp\left(-2\alpha m^{2}\right)}{Z^{2}}$$

After its substitution in Eq (11), we obtain

$$S=a\left(1+s\right),\;\;s\equiv \frac{1}{2aZ^{2}}\;\sum_{m\neq 0}\;\exp\left(-2\alpha m^{2}\right)\frac{k+\sin^{2}\left(2m\gamma H\tau \right)}{1+{\left(2m\gamma H\tau \right)}^{2}}$$ (12)

The above series can be simplified by using the integral representation of the sums: $Z=\sqrt{\pi /\alpha }$ and $a=k\sqrt{\alpha /8\pi }$.

To link *P*, which now reads *p* + *awτ*(1 + *s*), to an observable, we write the first-order kinetic equation for the number *N* of gyroscopes per unit of tissue volume, $\dot{N}=w-PN$. This gives *N* = *w*/*P* in stationery conditions. We would like to know the relative change of *N* under the HMF exposure, as MF drops from *H*_g_ to smaller values *H*. This is the relative number of gyroscopes *ρ* ≡ 1 − *N*/*N*_0_, where *N*_0_ = *w*/*P*_0_ is a solution for the geomagnetic field. Substituting *P* in *ρ* and *s* in *P*, and given *γH*_g_*τ* ≫ 1 and hence *s*(*H*_g_) ≈ 0, we finally arrive at the relation for observable *ρ* in HMF:

E(H)≡ρ(H)=1-11+(s(H)1+(τk/τ (13)

where *τ*_k_ ≡ *p*/(*aw*) is a kinetic time constant and *s*(*H*) is given by Eq (12).

**Fig 6:**
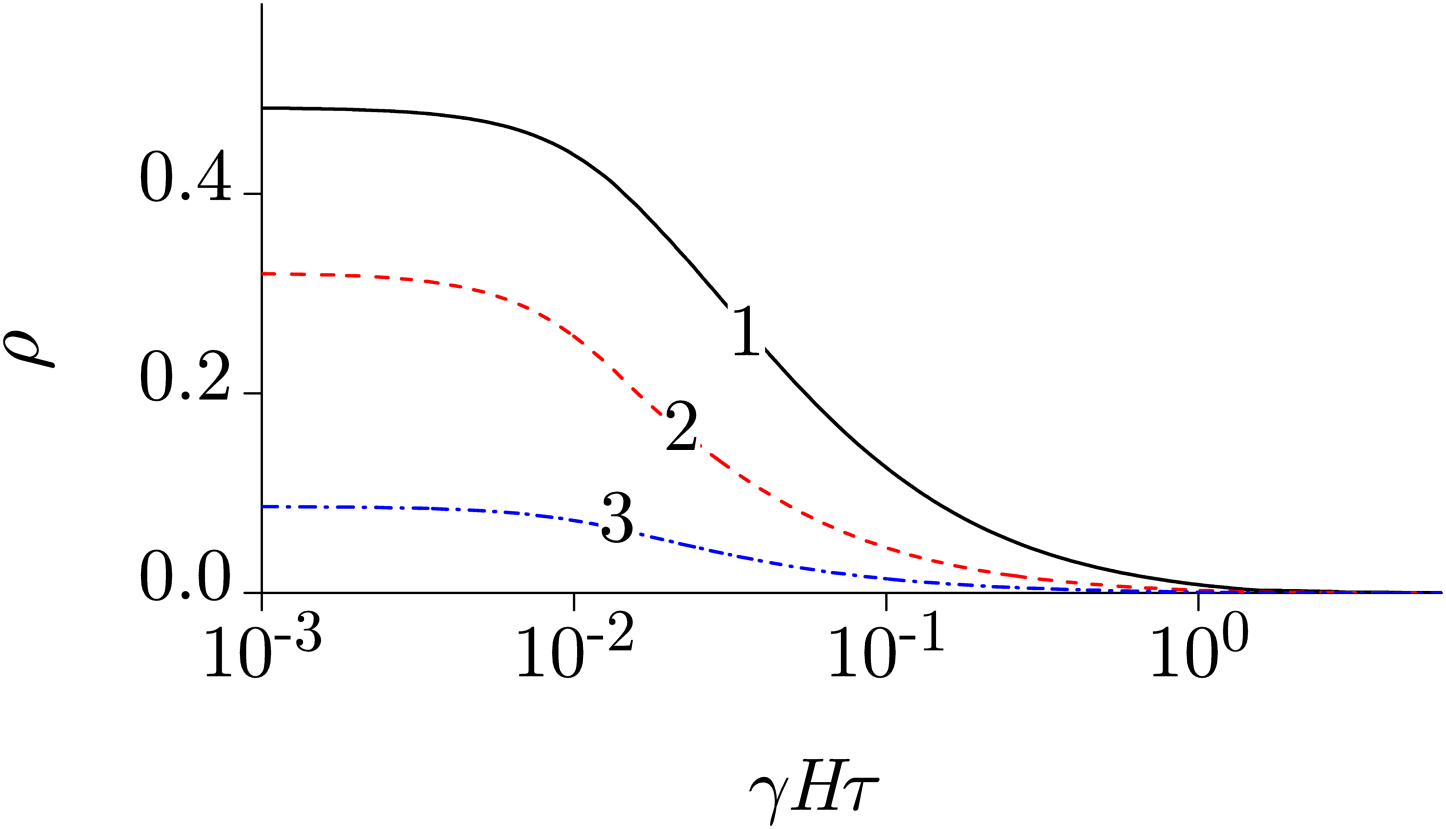
A magnetic vacuum effect. The number of molecular gyroscopes in HMF at different ratio of the thermal relaxation time and the characteristic time of chemical kinetics: *τ* ≫ *τ*_k_, *α* = 10^−4^ (1), *α* = 10^−5^ (2); and *τ* = 0.1*τ*_k_, *α* = 10^−4^ (3).

Function (13) is shown in Fig 6 for the case of *α* = 10^−4^ and 10^−5^ that corresponds in the order of magnitude to *I* ∼ 10^−37^ and *I* ∼ 10^−36^ g⋅cm^2^—moments of inertia of Glu residue at rotation around the long axis and the peptide bond respectively. As is seen, the critical MF is about 0.03/*γτ*.

Apparently, the quantum gyroscopic mechanism can explain biological effects of HMF, the relative magnitude of the magnetic effect reaching, theoretically, impressive 50% for small residues and about 10–15% for larger ones in the case of large thermal relaxation times. Recall that in the RPM, the electronic states lose their coherence in time intervals less than 10^−7^ s. In contrast, as calculated in [180], the rotational states of molecular gyroscopes can live milliseconds, reaching 100 ms for Glu-like object rotating in a cavity of radius 1.5 nm. As far as the gyromagnetic ratio is of the order of *e*/*Mc*, where *e* is the elementary charge and *M* is the mass of Glu, the critical MF for this case is about 0.5 *μ*T. Some difficulty is that the critical MF quickly grows for smaller cavities, becoming about 8 mT in a 1-nm cavity. On the other hand, this hypothetical mechanism is reinforced by the fact that quantum processes associated with whole large molecular fragments must play a crucial role in solving the problem of protein folding [207].

### Stable magnetic nanoparticles

Magnetic nanoparticles—tiny magnets—have been found in the body of many animals [208]. Often, magnetic nanoparticles in organisms are single-domain magnetite Fe_3_O_4_ crystals. Depending on their size, these particles are mainly divided into two classes: superparamagnetic (SP) and stable ferrimagnetic particles. The latter particles have a permanent magnetic moment that is rigidly bound to the particle’s geometry: the magnetic moment is said to be blocked. The magnetic moment of SP particles is not blocked, and is capable of changing its direction more or less randomly under the action of heat. In many studies, these facts are drawn to explain magnetic susceptibility of organisms, e.g. [30, 209].

We recall that mechanisms that could provide only a non-specific response to a magnetic stimulus are considered. For this reason, some proposed mechanisms are not commented on. For example, micrometer-sized ferromagnetic crystals, which could naturally grow in a few places in the beak of homing pigeons [28], are likely to exert a mechanical pressure on bird’s ophtalmic nerve [210]. However it is clear that regular occurrence of so large crystals would be an evolutionary formed property that provided a specific function in some species only.

#### Thermal equilibrium of stable nanoparticles

In MF *H*, a single-domain magnetite nanoparticle with a blocked magnetic moment *m* can rotate, reducing its magnetic energy *ε* = −**mH** = −*mH* cos *θ* where *θ* is the angle between the moment and the MF. If the particle is associated with a nearby biophysical structure, its turnings may initiate the cascade of subsequent events and cause an observable biological response. In the absence of MF and under the action of heat, the particle together with its magnetic moment experience chaotic turns. In the MF, some ordering occurs so that the magnetic moment is oriented in average in the direction of the MF, but continues fluctuating. The degree of ordering is an average value 〈*q*〉, where *q* ≡ cos *θ*. At high ordering, this value is close to unity, and to zero otherwise. In classical statistics, at thermal equilibrium, random variable *q* is subject to the Boltzmann distribution. Under these conditions, the degree of order is determined by the Langevin function L(*x*), e.g. [211] p. 83

$$\left\langle q\right\rangle =L\left(x\right)\equiv \coth x-1/x,\;\;x\equiv \frac{mH}{kT}$$ (14)

Since *q* is a random variable, one can find its variance, which proves to be equal to the derivative (denoted by accent) of the Langevin function:

$$\sigma_{q}^{2}=L^{\prime }\left(x\right)=\frac{1}{x^{2}}-\frac{1}{\sinh^{2}\left(x\right)}$$

It has been assumed in [209] that for magnetoreception to occur, an MF-induced change in the average value of the ordering should be greater than its standard thermal deviation: $\Delta \langle q\rangle >\sigma_{q}=\sqrt{L^{\prime }\left(x\right)}$. Since Δ〈*q*〉 = 〈*q*〉′Δ*x* = L′(*x*)Δ*x*, then condition $\Delta x\sqrt{L^{\prime }\left(x\right)}>1$ should de satisfied. The smallest Δ*x*, which means the highest sensitivity, is available for the largest value of $\sqrt{L^{\prime }\left(x\right)}$ equal to $1/\sqrt{3}$. That is, Δ*x* > 1.7. In other words, for the magnetic biological effect to be observable, the MF change Δ*H* should exceed 1.7 *kT*/*m*. The magnetic moment is *m* ∼ *vJ*, where *v* is the volume of nanoparticle and *J* = 480 G is the saturation magnetization of magnetite (CGS units). Then, for the ensemble of conditionally reference magnetite nanoparticles with the radius of 50 nm at physiological temperature, an MF change must be greater than about 30 *μ*T.

This mechanism is not applicable for interpretation of the magnetic navigation by animals, in which the latter show sensitivity at the level of tens of nT. For this reason, it has been assumed [209] that *N* = 10^6^–10^8^ magnetic nanoparticles each excite one nerve, while the brain performs an averaging summation of the input signals, so that the sensitivity of the entire system increases as $\sqrt{N}$, in accordance with the central limit theorem of mathematical statistics. However, this hypothesis yet remains unapproved. Moreover, this is not consistent with the reliably established fact of magnetic sensitivity in organisms, in which there is neither a large number of nanoparticles nor a nervous system.

Note that the need for the hypothesis of large *N* is the result of a general flaw of this approach in explaining magnetoreception. The flaw is that the Langevin formula is used under conditions, in which it is not justified. The Langevin formula assumes a statistical averaging. In the above case, the averaging is obviously carried out at the stage that precedes the chemical processes. Thus, it is implicitly assumed that each nanoparticle, before initiating a subsequent chemical event, mysteriously behaves in an averaged manner, i.e., similar to the whole statistical ensemble of such particles. Evidently, this is not the case. Instead of averaging the state of nanoparticles, one should statistically average kinetics of the subsequent chemical events. Then one does not have to suggest the involvement of a vast number of nanoparticles in magnetoreception, because a single particle in its individual behavior can induce a chemical event.

We add that in low MFs, the magnitude of the effect that is associated with value 〈*q*〉, is proportional to *H* (Curie’s law), and hence there is no a rapid change in 〈*q*〉 at *H* decreasing from GMF to 0. Obviously, this mechanism does not apply to explain the zero-MF effects when the magnitude of the effect is experiencing an abrupt change in MFs of the order of 1 *μ*T or less.

#### Stable nanoparticles in double-well potential

Single magnetoreceptors that are highly sensitive to the MF variations on the background of the geomagnetic field have been proposed in [212]. In this model, a nanoparticle with blocked magnetic moment is in a rotational potential created by the cytoskeleton filaments and the MF. In general case, this potential is composed of two wells separated by a barrier. The MF can change the height of the barrier. The particle undergoes stochastic Brownian rotations under the influence of thermal perturbations and is able to overcome the barrier. The transition probability depends exponentially on the barrier height. For this reason, even small MF variations might influence the rate of transitions and, thereby, cause a biological response. The minimal level of such variations is calculated to be of the order of 100–200 nT in a reference nanoparticle.

What are the properties of this model in the zero MF mode, when *H* → 0?

**Fig 7:**
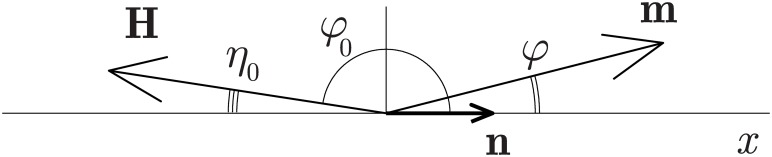
Notation for variables. Relative position of the MF vector **H** and the magnetic moment **m** of a nanoparticle. Note that **m** cannot follow **H** where *φ*_0_ is about *π* as far as a deviation of **m** from direction **n** produces a restoring torque of magnitude −*κφ*; hence, two potential minima occur over *φ*.

In general, the stationary orientation of the nanoparticle magnetic moment does not follow the direction of the constant MF. This orientation is determined by the balance of the elastic torque of cytoskeleton filaments and that of magnetic forces. Let *x* axis determines the equilibrium direction of the magnetic moment of the particle in the absence of MF and thermal disturbances, Fig 7.

**Fig 8:**
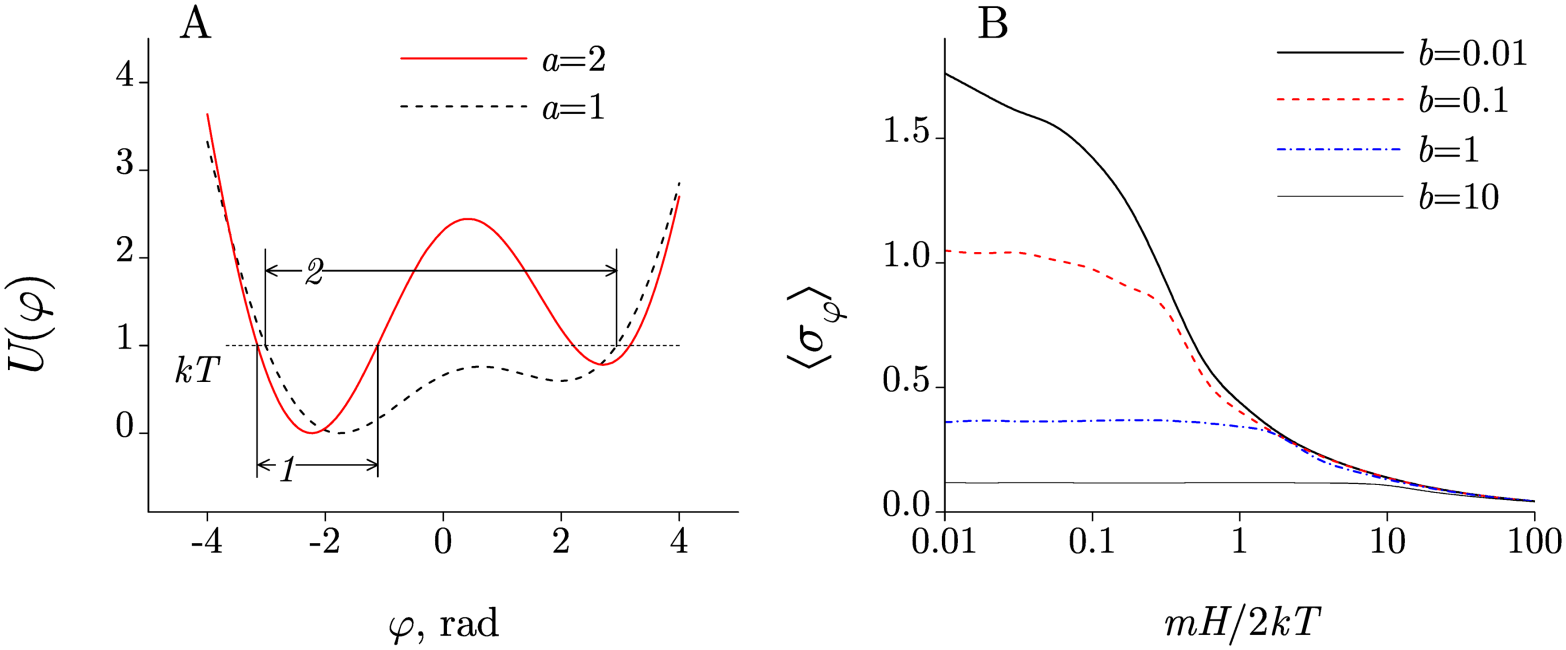
Characteristics of the random rotational oscillations of a magnetic nanoparticle. (A) The potential function of the particle with different values of the parameter *a* = *mH*/2*kT* that is proportional to the MF, *b* = 0.5, and *φ*_0_ = 1.1*π*. (B) The averaged standard deviation of the particle oscillations under the action of thermal disturbances at different values of the elasticity of the cytoskeleton *b* = *κ*/2*kT*.

Rotational potential of the nanoparticle then equals

$$U\left(\varphi ,\varphi_{0}\right)=\kappa \varphi^{2}/2-mH\cos\left(\varphi -\varphi_{0}\right)$$

where *φ* is the angle of particle (or its magnetic moment) deviation from the equilibrium direction *x*, *κ* is the cytoskeleton elasticity coefficient, *φ*_0_ is the angle between the MF and the equilibrium direction. For particles that are mainly oriented against the direction of the constant MF, i.e., when *φ*_0_ ∼ *π* (or *η*_0_ ∼ 0), the potential has a double-well form, Fig 8A.

Further, we assume that nanoparticles are mobile enough to significantly change their orientation in the geomagnetic field under the heat. In other words, mechanical elasticity *κ*, associated with the bending of the filaments, magnetic elasticity *mH*, and the scale of the thermal perturbations *kT* differ within one–two orders of magnitude only. Furthermore, we assume that each nanoparticle is fixed in such a manner that it can mainly revolve around a single axis.

Fig 8A shows that in some MF *H* = 4*kT*/*m*, or at *a* ≡ *mH*/2*kT* = 2, the particle motion in the lower well occurs within a narrow range *1* that is determined by the width of the well at the level *kT*, while a reduction of the MF to 2*kT*/*m* leads to a significant expansion of the area of possible motion to the size *2*.

The average amplitude of the oscillations within the well can be defined as twice the standard deviation of a particle moving in a viscous medium under the influence of thermal disturbances. The Langevin equation for the rotation angle of a particle is

$$\gamma \frac{d}{dt}\varphi \left(t\right)-U_{\varphi }^{\prime }\left(\varphi ,\varphi_{0}\right)=\sqrt{2\gamma kT}\;\xi \left(t\right)$$

where *ξ*(*t*) is a random moment with the correlation function 〈*ξ*(*t*)*ξ*(*t* + *τ*)〉 = *δ*(*τ*) and *δ*(*τ*) is the Dirac delta-function. By means of substitutions *a* = *mH*/2*kT*, *b* = *κ*/2*kT*, and *t* = *t*′*γ*/2*kT* it can be reduced to a dimensionless form $\dot{\varphi }-U_{\varphi }^{\prime }=\xi$, where $U_{\varphi }^{\prime }=b\varphi +a\sin\left(\varphi -\varphi_{0}\right)$, and can be integrated numerically, for example, by the Runge-Kutta method. Results are shown in Fig 8B.

Shown are dependences of the standard deviation, averaged over the uniformly distributed angle *φ*_0_, on the parameter *a* = *mH*/2*kT* for various *b*. It is seen that the scope of random rotations increases significantly with decreasing MF, especially for the least fixed particles. Increasing of the amplitude of random oscillations takes place in a logarithmically wide range of parameters.

In other words, in the geomagnetic field, a significant portion of nanoparticles are “locked” in one of the two narrow potential wells. After reducing the level of the MF, the potential barrier between the wells disappears, and the particles under the heat begin rotating more freely. We recall that larger oscillations supposedly cause the subsequent downstream biophysical and biological effects. In other words, in this model, the magnetic effect is associated with the magnitude of Brownian rotational deviations

$$E\left(H\right)∼\left\langle \sigma_{\varphi }\right\rangle$$ (15)

This MF-dependence is shown in Fig 8B.

Nanoparticles produce their own MF. In their vicinity, this MF exceeds the geomagnetic field by several orders [31]. In the zero MF, the increase in oscillations of the large own MF could influence the biradical reactions. In contrast to the direct action of the zero MF on biradical reactions in organisms, the indirect action through the rotations of nanoparticles does not require the assumption on the incredibly long lifetime of the spin-correlated biradicals.

Indeed, a small enough, but still stable particle, in a HMF, rotates about *π*/2 or larger, under thermal perturbation. Because of the great nonuniformity of the particle’s MF, a local MF seen by a radical pair can fluctuate in a few mT as the particle rotates, which is enough to shift the RP reaction together with the MF fluctuations. If the external MF grew to GMF, the same particle would not rotate, there would be only unchanged local MF near the RP, and no signal would propagate from it. This means that in the presence of magnetic nanoparticles, MF switching between GMF and HMF would cause a fluctuating biochemical signal through the adjacent RP reaction. Unlike this, if no magnetic particle existed, the RP could only see that the external MF changed from HMF to GMF, rather than in a few mT. Note that the RP could detect GMF to HMF changes in this case, however this would require the electron pair to be a slowly thermally relaxed object, which is mostly impossible, as shown in Section “Radical pair mechanism.”

Returning to the alternative hypothesis based on stable magnetic particles, one should notice, nevertheless, that numerical values for the critical MF

H∼2kT/m (16)

are not promising for the HMF effect to be explained. In order to explain the HMF effect, the deflection point in Fig 8B should correspond to about 1 *μ*T or less. This required value can be gained for the particles of radius greater than 160 nm, which is well above 70 nm—the size where a magnetite particle becomes a multidomain one and significantly reduces its magnetic moment. For a reference particle of radius 50 nm, the critical field, as we saw above, is about 30 *μ*T.

Thus, the rotational dynamics of blocked single-domain nanoparticles, while remaining suitable for detecting small geomagnetic variations [212], could not be involved into the magnetic vacuum effect.

### Superparamagnetic nanoparticles

SP nanoparticles can occur in the body due to oxidation of ferrihydrites in ferritin protein and subsequent crystallization of iron oxides [213]. Depending on the species and individual characteristics of the organism, these processes may have a status from normal to abnormal and be accompanied by the appearance of significant amounts of SP nanoparticles. Magnetite/maghemite concentration in human tissues varies from tens to hundreds ng per gram [214]. The equilibrium density and distribution of SP nanoparticles in tissues are not yet known in those organisms that do not possess specific magnetoreception.

Some possibilities to explain the HMF biological effect could be associated with SP particles. There are a few modes of the magnetization state in which one could examine the effect of MF.

#### Thermal equilibrium of SP particles

Superparamagnetism occurs in nanoparticles where the thermal energy *kT* is higher than that of magnetic anisotropy of the particles. It happens in a small enough particle because anisotropy energy is proportionate to the volume of particle. Then, magnetization can spontaneously switch among different directions similar to that in paramagnetics, the Langevin theory being adequate. However, as we saw in Section “Thermal equilibrium of stable nanoparticles,” the critical MF for this mode is about 2*kT*/*vJ*. In the case of magnetite Fe_3_O_4_ at 37°C, particles of radius less than about 10–12 nm are superparamagnetic (see below). Consequently, for these particles, the critical MF would be higher than about 1 mT. This makes a hypothesis of HMF megnetoreception based on thermal equilibrium of SP particles unworkable in explaining HMF effects.

#### Magnetostatic interaction of SP clusters

It was assumed that SP particles concentrated near the trigeminal nerve in the upper beak of some birds aggregate into interacting clusters. Depending on the direction of the external MF, the clusters can attract or repel each other, thus providing a mechanical effect on adjacent biophysical structures [215]. Such structures can be, for example, nerve cell membranes that contain mechanosensitive ion channels. The following analysis shows, however, that this mechanism does not work.

The magnetic moment of an idealized spherical particle depends on its radius *r* and saturation magnetization *J*: *m* = (4/3)*πr*^3^*J*. Energy *mH* of the SP magnetite particles in the geomagnetic field is much smaller than *kT*. Therefore, *x* ≡ *mH*/*kT* ≪ 1, and the Langevin Function (14) is L(*x*) ≈ *x*/3. Accordingly, the magnetic moment of the cluster is determined by the Curie law: *μ* = *mN*L(*x*) ≈ *mNx*/3. Here, *N* is the number of particles in the cluster, *N* ∼ (*R*/*r*)^3^ ∼ 10^5^, where *R* is the cluster radius (see below).

In the order of magnitude, the energy of interaction of two clusters at a distance *d* is

$$\varepsilon ∼\frac{\mu^{2}}{d^{3}}=\frac{m^{2}N^{2}x^{2}}{9\;d^{3}}$$

Under MF variations, this energy is transformed into the potential energy of compression or expansion of a biological tissue and is distributed—which was not addressed in the work [215]—over many degrees of freedom. Their number is obviously on the order of the number *n* of the valence bonds in the area that is exposed to the strain between the two clusters. Numerically, *n* ∼ *dR*^2^ *ρ*, where *ρ* (in this section) is a bulk density of the valence bonds in protein structures.

For possible reception of the MF variations, the energy change per degree of freedom should be on the order of *kT* = 4.14 × 10^−14^ erg or more. Using the above expression for *ε*, *x*, *N*, and *n*, we derive that the minimum MF detectable in this way is

$$H∼\frac{d^{2}{\left(kT\right)}^{3/2}\sqrt{\rho }}{\pi^{2}J^{2}R^{2}r^{3}}$$

This quantity can be easily evaluated. The measured radius *R* of the clusters was about 0.2–0.4 *μ*m and distance *d* was 1 to 2 *μ*m. Substituting the value of *ρ* ∼ 10^23^ cm^−3^, *J* = 480 G, and *r* ∼ 5 nm for the particle radius in the clusters found in the beak of domestic pigeons, we finally obtain *H* ∼ 5 × 10^4^ G, or 5 T. Such a large value of the minimum detectable MF invalidates this scenario. With respect to the zero-MF effects, it apparently also has no future.

#### MF influence on the barrier height

In larger particles, the anisotropy energy that is minimal in a few directions begins to play a role, and static and dynamic effects could be also examined.

In the dynamics of stable single-domain nanoparticles, a factor that made HMF effect possible was a rotational potential barrier, the height of which depended on the MF magnitude, see Fig 8A. SP particle dynamics is similarly related to overcoming a barrier. When the external MF is zero the magnetic moment **m** of a nanoparticle in the state of equilibrium is directed in parallel to one of the so-called easy magnetization axes—along or against an axis *x*, in the case of uniaxial anisotropy. The transition between these states is associated with a deviation from equilibrium. This is accompanied by an increase in the magnetic anisotropy energy that is equal, in the simplest case, to *Kv* sin^2^ *φ*, where *φ* is the angle between the magnetic moment and the axis *x*, *K* is the uniaxial magnetic anisotropy constant, and *v* is the volume of the particle. In the external MF **H** directed along *x*, the particle potential takes the form

$$U\left(\varphi \right)=Kv\sin^{2}\varphi -mH\cos\varphi$$ (17)

In the interval *φ* ∈ [0, 2*π*), the potential has one minimum in large MFs, and two minima in small MFs. The barrier height is

δU=Kv-mH

with a critical MF—where the barrier height is zero—on the order of *Kv*/*m*, or *K*/*J*. For bulk magnetite *K* ≈ 1.35 × 10^5^ erg/cm^3^, e.g. [216]. For nanoparticles, the effective anisotropy constant can differ and be even an order of magnitude higher, mainly due to surface effects. For particles of radius 13 nm, *K* ∼ 3.4 × 10^4^ erg/cm^3^ [217], and for particles with radius 2 nm, *K* ∼ 1.2 × 10^6^ erg/cm^3^ [218]. We use the value for bulk magnetite.

This shows that the critical field *K*/*J* exceeds 280 G, or 28 mT. Therefore, MFs that are usual in magnetobiology cannot significantly change the height of the barrier, and the mechanism that was discussed in Section “Stable nanoparticles in double-well potential” does not work. MF, however, could affect the dynamics of a magnetic moment that flips between “easy” directions because the flip frequency depends exponentially on the barrier height.

#### Dynamics of SP magnetic moments

Provided *δU* ≈ *Kv* > *kT*, which is valid in our case for particles larger than a few nanometers, the rate of thermally activated flips in weak MFs is determined by the Neel relaxation time

$$\tau_{N}=\tau_{0}\exp(\frac{\delta U}{kT})$$

where *τ*_0_ is usually of the order of 10^−9^–10^−10^ s, e.g. [219] for fine particles and only weakly dependent on *δU* [220]. Calculating *τ*_N_ for idealized spherical particles of different radii with *δU* = *Kv*(*r*), one can find that there is a narrow range of radii, 10–12 nm, for which the relaxation times fall in the interval 0.01–100 s typical to most biological time scales. Larger particles are in blocked state, for which magnetic effects are possible and considered above. Smaller particles are in pure SP state, where no magnetic effects are expected as discussed above.

For particles of that intermediate size, one can estimate the relative magnitude of the changes in relaxation time under the MF change of Δ*H*,

$$E\equiv \frac{1}{\tau_{N}}\frac{d\tau_{N}}{dH}\Delta H$$

It is not difficult to find that for these particles, a MF change of 1 *μ*T causes a change in the value of the relaxation time of less than 0.08%.

As we saw above, stable single-domain particles increase the amplitude of the Brownian rotations with decreasing MF value, which can trigger a cascade of subsequent biophysical/biochemical processes and cause an observed biological response. In case of SP nanoparticles, not the particle itself but its magnetization vector experiences spontaneous rotations, which results in similar MF random fluctuations near the particles. As in the case of stable particles, these MF fluctuations are much higher than the geomagnetic field and are able to significantly shift the speed of nearby biradical reactions, thus leading to a biological response [31]. However, there are many factors unfavorable for possible HMF magnetoreception via SP nanoparticles: a narrow range of the sizes of nanoparticles that could respond to MF; the absence of a particular MF-dependence in the zero MF, as far as *mH* ≪ *Kv*; the lack of clarity how the changes in relaxation time could affect the downstream biophysical/biochemical events; and a small size of the effect—less than a tenth of a percent—under exposure to a characteristic HMF of the order of 1 *μ*T.

Apparently, the totality of these factors makes HMF magnetoreception by means of SP nanoparticles devoid of prospects.

### Protons in water

In a series of recent studies made by the group of A. Konovalov and reviewed in [151], there have been investigated a number of physical and chemical properties of aqueous solutions of different chemical compounds, for example, phenosan and *α*-tocopherol [136]. Studied were dependences of the properties on the concentration of dissolved substances in the range of 10^−20^–10^−2^ M. Physico-chemical properties of solutions in these low concentrations changed significantly with deprivation of the geomagnetic field. Biological properties, i.e., the effect of these solutions on some organisms, were also dependent on the preliminary exposure of the solutions to HMFs. These data, together with previous results of other authors, e.g. [221–224], reinforce the assumption that a possible mechanism of biological action of weak MFs comprises the intermediate stage of modifying the properties of the aqueous medium in cells.

Not all of the physical properties of water are determined by that undoubted fact that liquid water is a collection of water molecules. For example, water conducts electricity as a result of the dissociation of water molecules into hydroxyl and hydronium ions. Due to the specific Grotthuss mechanism of their mobility, these ions are called ionic defects in the structure of water. In addition to ionic defects, there are so-called Bjerrum’s orientation defects. All these defects break a structure rule according to which every oxygen atom covalently binds two hydrogen atoms, or protons, and every proton binds two oxygen atoms by means of a hydrogen bond.

From the viewpoint of electrokinetic properties, water is a collection of the defects that move by means of jumps of protons. Protons detach from their water molecules and form to some extent an independent system—a proton subsystem of water.

The proton subsystem of water is attractive in explaining nonspecific magnetoreception and searching the molecular target of weak MFs. There are the following reasons for that.

The spin of a proton is the same as that of an electron. Electron spins play a crucial role in chemical reactions by participating in a quantum exchange interaction. The exchange interaction of protons is much smaller in magnitude than that of electrons, but it could influence the mobility of water structure defects and contribute to the kinetic phenomena in aqueous solutions.

A proton has a magnetic moment associated with its spin. The impact of the MF on the magnetic moment means some control over the proton spin and, therefore, over the progress of the chemical process with the participation of the proton, provided the exchange interaction is of sufficient magnitude. The effect of proton exchange interaction on proton transport is a new water related mechanism to be considered.

As is known, a water molecule occurs in two isomeric forms. A water molecule can be in the ortho or para state, depending on the relative orientation of the spins of its protons—unidirectional or opposite-directional, respectively. In the gas phase, the equilibrium amounts of these isomers are at the ratio of 3:1, due to quantum mechanical considerations. Studies [222] and [224] show that liquid water can produce non-equilibrium amounts of water spin isomers in a significantly different ratio. This indicates that in liquid water, a metastable local spin ordering exists that can be magnetically sensitive by virtue of the connection between spin and magnetic moment.

Unlike free radical pairs, that are conjectured to be a possible MF target also in nonspecific magnetoreception, the number of protons in biological water medium is enormous, and they participate in many biochemical reactions.

It is essential that the de Broglie wavelength of protons in liquid water at room temperature is 2.5 Å, while their jump length is about 0.8 Å. So a proton in these processes is a quantum object and should be described by the wave function.

**Fig 9:**
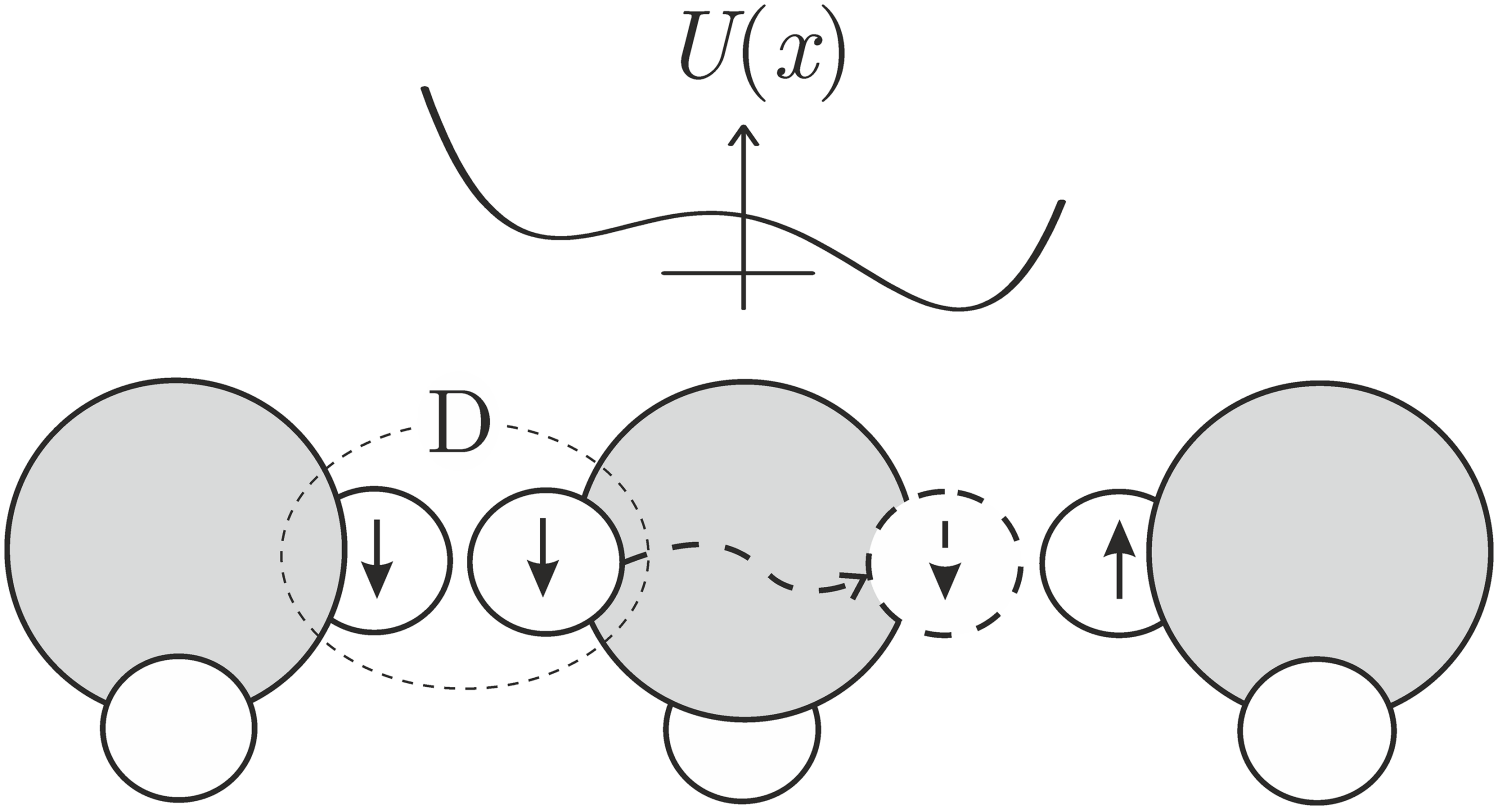
Motion of a D-defect due to the tunneling of a proton to the neighboring H-bond along the reaction coordinate *x*. White circles are protons, gray circles are oxygens. Shown are the exchange interaction potential *U*(*x*) of the tunneling proton and proton spin states.

Spin effects in proton tunneling can occur due to the proton wave functions covering the nearby potential wells in the network of hydrogen bonds. If a neighboring well is empty, the tail of the wave function that covers it, determines the probability of a quantum transition of the proton into this well. However, if the neighboring well is occupied by another proton, e.g., as in the orientation defect of D-type in Fig 9, then, due to the overlapping of the wave functions of two protons, the exchange interaction occurs between them, which depends on the mutual orientation of their spins. The exchange interaction leads to a coupling between the coordinate of the proton and the spin state of the system. The coupling can be controlled by an external magnetic field through the proton magnetic moments. Thus, probability of the proton jumps between wells varies. Recent studies [225] show that many electrical, dielectric, and even chemical properties of water can be explained by proton jumps rather than water molecule rotations.

In addition to the interaction of protons and electrons, which forms hydrogen bonds, the protons react with each other. The interaction of protons, the Coulomb repulsion, includes as part the exchange energy that depends on the relative orientation of spins.

Most of the hydrogen bonds in water contain only one proton. To describe the orientation defects in the grid of hydrogen bonds, it is assumed that a part of the bonds contain two protons to form so-called D-defects, Fig 9, or do not contain them at all—L-defect.

Fig 9 shows a proton potential along the reaction coordinate—when moving between bonds with different spin orientations of their protons. The potential is asymmetric for the spins in opposite orientation. If the asymmetry is large enough, then a jump to the well with higher energy may not occur until the well is occupied by a proton in the suitable spin state. Evidently, this impedes relaxation of the local spin equilibrium and affects the mobility of water structure defects.

Thus, the wave functions of two protons that are localized in adjacent wells of a D-defect overlap providing their exchange interaction. Since the MF affects spin magnetic moments, it affects the exchange interaction, and hence the mobility of structural defects. Mobility of defects, in turn, contributes to the proton activity in biochemical reactions.

The proposed protonic mechanism of magnetoreception looks like that kind of the biradical mechanism, in which the evolution of electron spins—the singlet-triplet transitions—is affected by the MF of the magnetic moment of a third particle, such as a proton. In the protonic mechanism, all three particles are protons, and the external MF affects the evolution of the whole system.

Most protons in water solutions relax thermally very slowly, in a matter of seconds; so even a weak ac MF *h* can significantly change the state of these protons. An estimate of the ac MF in the order of magnitude can be obtained from a consistency between the inverse value of proton relaxation time *τ*_p_ and the Rabi frequency *γ*_p_*h*—the frequency with which the projection of spin on a constant MF changes, where *γ*_p_ is the proton gyromagnetic ratio. Hence, at a resonance frequency, the MF amplitude that can overturn a spin during its life time is *h* ∼ 1/*γ*_p_*τ*_p_ ∼ 1 nT. On the other hand, we saw that condition *γHτ* ∼ 1 plays an important role in HMF magnetoreception and determines the level of dc MF, in the order of magnitude, at which the HMF effect is possible: *H* ∼ 1/*γτ*. Thus, in the RP mechanism, this level 1/*γ*_e_*τ*_e_ at *τ*_e_ ∼ 10^−7^ s is 57 *μ*T and is too large to explain the HMF effect. Spin relaxation time of water protons is about 3 seconds, which again gives

$$H∼1/\gamma_{p}\tau_{p}∼1\;\text{nT}$$ (18)

This, of course, does not mean that changes in the spin subsystem must be accompanied by readily observable effects. A great relaxation time means a feeble link of spin subsystem with molecular vibrations, which in turn makes it difficult to convert the MF signal into the state of the molecules responsible for chemical reactions.

We note that the energy of the MF interaction with the proton magnetic moment is rather small, of the order of 10^−10^ *kT* (that does not dismiss the RPM). On the other hand, due to the rigid connection of spin and magnetic moment, this weak magnetic interaction controls the relatively strong exchange interaction that can even be greater than *kT* for weakly bound protons [181]. In other words, the MF affects the probability of proton jumps by means of the Pauli exclusion principle; this modifies the mobility of water structure defects, and possibly modulates the rate of some biochemical reactions.

## Discussion

The main objective of laboratory magnetobiology is to determine biochemical signaling agents, or messengers, involved in magnetoreception in its very first stages. Some information about the molecular nature of magnetoreception can be derived from behavioral reactions. However, among measured values, the major ones are the concentrations of various substances in organisms and their rates of change.

In the laboratory magnetobiology, two experimental approaches can be distinguished with regard to the identification of the primary biochemical messengers. It is (i) an indirect biochemical search of the “primary” molecules that respond to a change in the magnetic environment, and (ii) a direct identification of these molecules based on their gyromagnetic factor from the MF-dependences of easily detectable values, such as the concentrations of second messengers. Among the approaches of the first type, for example, are the search for genes that are “responsible” for magnetoreception, cytological studies of the brain slices before and after a magnetic exposure, and others. It is a widely used approach in modern magnetoreception studies. However, with these indirect methods, it always remains unclear whether a change in the concentration of molecules found is a cause of biological reactions or, instead, an effect of the changes in some other molecules.

The search for microscopic targets directly from the MF-dependences requires complicated physical facilities and numerous measurements with a sequential variation of the MF parameters. It is an expensive and time consuming approach. For this reason, it is represented by a smaller number of studies. However, it is this method that is able to provide the most compelling evidence of the molecular processes.

It is worth noting that researchers who use such different approaches, give as well a completely different meaning to the concept “magnetoreception mechanism.” For the followers of the direct method, the concept of mechanism includes the fundamental physical stage of the MF interaction with elementary particles or atoms and ends with the identification of biophysical/biochemical process associated with those particles. For the followers of the indirect method, the magnetoreception mechanism only begins at this latter level and further includes a determination of the complex biochemical pathways of a magnetic signal transduction to the level of downstream organismic reactions.

If we have in mind only the primary fundamental physical mechanism, the experimental data of the first part of this review and the observed lack of any correlation among them are not neutral with regard to identifying the primary mechanism. They provide a guidance on the possible physical properties of the molecular MF target.

First, HMF effects are very similar to the effects of weak variable MFs. There is a wide range of the magnitudes of the effects that reveal themselves at all levels of organization of living systems, from bacteria to humans. Biological characteristics in their varied forms are subject to changes. Functional or medical records, where applicable, can both worsen and improve. Maximum sensitivity to the magnetic factor occurs in the early stages of development and in the periods of cell differentiation. There is a high sensitivity to the physical, chemical, and physiological conditions, as well as low reproducibility. Leaving aside the effects of stronger MFs, the similarity of the effects of HMF and weak ac/dc MFs indicates their common nature, a common primary physical mechanism of magnetoreception.

Second, there is a great variety of effects observed both in HMFs and in other MFs. It has not yet been possible to establish any common conditions controlling the appearance of the magnetic effects in different organisms or populations rather than in their individual forms. There is no correlation of the HMF effect magnitudes with the HMF values and the durations of HMF exposure, in a set of the experiments with different biological species. This suggests that there is no specific MF targets in organisms, with the possible exception of the evolutionarily created magnetic sense in some migrants.

At various times, different molecular objects have been declared to be a specific target, e.g., ion channels, calmodulin, magnetic nanoparticles, and flavine-tryptophan biradicals in cryptochromes. However, there is still no clarity. Apparently, the very absence of a pattern in nonspecific magnetoreception becomes a kind of regularity that needs to be explained. We believe that the MF targets in organisms do not possess a biological specificity. The targets are likely to be physical objects that are widely distributed over various biophysical structures. Only through a confluence of random physicochemical circumstances, a particular group of targets become capable of acting on the associated specific biological messengers and cause an MF-mediated downstream reaction. Effects due to the fundamental interaction with magnetic moments result in all possible effects at the primary molecular level, but only a small part of them become measurable at higher levels due to the concurrence of various circumstances. It is very different from the effects of chemical agents like, for example, snake venom or an opioid antagonist/agonist that always have their specific molecular targets.

### Comparison of the HMF effect models

Several mechanisms could qualify for an explanation of the effects of non-specific magnetoreception. Table 2 presents their properties: type of the model—quantum mechanical or within classical dynamics, a formula for the critical MF, its expected value, and a link to the corresponding equation in the article.

**Table 2:** Putative mechanisms of nonspecific magnetoreception.

Mechanism	Model	Relation	Crit. MF	Equation
Radical pair mechanism	QM	*γ*_e_*Hτ*_e_ ∼ 2	1 mT	(4)
Magnetite-based mechanism	C	*mH*/2*kT* ∼ 1	30 *μ*T	(16)
Universal physical mechanism	C	*γHτ* ∼ 3	*	(6)
Molecular gyroscope mechanism	QM	*γHτ* ∼ 0.03	*	(13)
Water proton mechanism	QM	*γ*_p_*Hτ*_p_ ∼ 1	1 nT	(18)

* Critical MF can be determined from experiment.

It can be seen that the first two mechanisms—widely discussed in the literature the radical pair and the magnetite-based mechanisms—are unlikely to explain the HMF effects. The critical MF, where a magnetic response could be expected, is too large as compared with the experimentally established one of the order of a *μ*T. Due to the common nature of the HMF effects and those of ac MFs of about *H*_g_, these mechanisms are also unlikely with regard to the ac MF effects that show frequency selectivity.

Two mechanisms of nonspecific magnetoreception are more likely: the universal physical and the gyroscopic mechanisms.

The universal, or general physical, mechanism is interesting because its predictions do not depend on the nature of the molecular magnetic moments. It is assumed only that they precess and relax. Therefore, there is an attractive possibility to measure the MF target parameters, the gyromagnetic ratio and the thermal relaxation time, separately. The mechanism predicts the maximum effect of approximately 12% and the existence of HMF and ac/dc MF effects simultaneously in one and the same organism.

The magnetic moment of a molecular gyroscope does not precess. The moment occurs due to the rotation of charges that are distributed over the gyroscope—a rotating molecule with its ends temporarily fixed. Gyroscopic mechanism is attractive by the high value of the expected effects, up to 50%, and a relatively small value, 0.03, of the critical parameter *γHτ*. However, (i) the critical MF is highly dependent on the size of a virtual space for rotation, and (ii) the existence of the long-, or coherently, rotating parts of a protein chain in the process of folding has not yet been confirmed in any experiments besides magnetobiological ones.

Is it possible to distinguish between the precession and gyroscopic mechanisms in the experiment? It is not clear yet. Perhaps, for this to be done, one needs first to determine the parameters *γ* and *τ* of the MF target. As shown in [189, 226], molecular mechanisms are sensitive to the rotation of samples. Any pequliarity in *H*-dependence should shift proportionally to the speed of rotation and inversely proportionally to the gyromagnetic ratio.

The proton-exchange mechanism requires very precise technique for its validation—a magnetic exposure system that could eliminate any extraneous MF variations exceeding 1 nT. Available observations of 10 to 1000 greater critical MFs do not support the mechanism. In addition, this has not been mathematically developed to a level that would allow one to make experimentally verifiable predictions. Further, even if the mobility of a portion of water protons was really changed in 1-nT MFs, it is unclear how this change could affect the rate of biochemical reaction.

Besides all listed above, there are no other microscopic magnetic moments to consider that could be involved in magnetoreception.

### Methodological comments

As follows from the data provided by the table column “Questions,” many articles contain certain omissions that impede unambiguous interpretation of the results. It is useful therefore to formulate some general methodological comments that could improve the situation in the future.

Currently, there are about two hundred publications, documenting the fact that the magnetic vacuum causes a reaction of the organisms. However, the MF-dependence of the effect has not yet been established. There were only a few studies that investigated dependences of the biological effects on the MF value with its *gradual* decrease, and this does not allow one to compare experiments with theory fruitfully. At the same time, there is a theory exposed in Section “Universal physical mechanism” that predicts the kind of the functional MF-dependence and provides the method of extracting information about the nature of magnetic targets from such dependencies. Under these conditions, one could expect new experimental works that fill the gap. It is important to pay attention to the logarithmic character of the expected dependency, see e.g. Fig 4A, and thus the need in a logarithmic MF increment in the experiment. The number of points in the MF-dependence of a measured parameter should provide stability of interpolation. Another significant methodological factor of the experimental design is the mode by which the MF is switched on or off. If such a switch is simply a closure of a circuit, which starts or stops flowing current, it is accompanied by a rapid change in the magnetic field. In turn, a pulse of the induced electric field and, accordingly, an electric current pulse in a test sample tissue occurs. For standard laboratory systems of the magnetic exposure, this current may be greater than the biologically endogenous currents by several orders of magnitude, thus causing a biological response with an aftereffect. Then the response will be mistakenly associated with the MF action. This factor of nonreproducibility can be easily removed by smoothing the MF switching process, which will preclude the emergence of uncontrolled current pulses.

Table 1 shows that only two types of exposure systems are used in the study of the biological effects of HMF: magnetic shielding and passive compensators of the GMF. Both methods have advantages and disadvantages that have not been properly addressed in almost all reports.

For compensation the advantages are that the non-electromagnetic environment is not disturbed so that the interference of other senses such as sight and smell are preserved between exposure and sham conditions. However in almost all cases the compensations are fixed in time so do not correct for variations in the ambient static and ELF magnetic field. Also there is no correction for confounding effects of electric fields and RF fields, what is crucial for obtaining reproducibility in different laboratories, where such fields may vary significantly. For example, the recent work [12] indicates that the animal orientation effects of a static geomagnetic field can be disturbed by effects of man made RF noise. It could be better if in this compensation approach, systems capable of active correct were used; however this technology is limited to relatively low frequencies and would not address the RF issue.

For the shielding approach it is important to have a number of controls. It is necessary to correctly address electrical, thermal, acoustic, humidity, and lighting conditions. A sham shielded container that affected light and smell but did not attenuate any ambient EM radiation is needed. A second control container is needed that only shields electric fields “identical” to the electric field shielded in the experimental container. Then if light is to be introduced, it must be done so such that there is no increase in temperature or introduction of EM fields. Finally if magnetic fields are introduced as suggested in testing the universal mechanism hypothesis, then they have to be introduced in such a way that they do not introduce electric fields and are not distorted by proximity to the shielding material. Such care in the shielding experiments have been reported [4] but are rare.

The selection of compensation versus shielding should also be matched to the question being investigated. For example, if one wanted to look at the effects of HMF in space travel, the environment of both the sham and exposure systems should reflect as much as possible other unique exposure conditions such as light, sound, electric fields, and RF fields. Since these confounds are bound to be very different then in the laboratory on earth doing experiments in a shielded facility and then introducing the other stimuli may be the best approach. Of course, a major confound would be the presence of gravity.

Not infrequently, the articles provide no information on the background variable electromagnetic fields and slow variations of the GMF. However, such fields can significantly alter the magnitude of the observed biological effects.

Sometimes, researchers comment on the effects of weak MFs, less than 1 *μ*T, based on the results obtained from experiments with a relatively strong MF of the order of mT and more. Since the molecular mechanisms of the action of weak and strong MFs can be substantially different, such comments are not convincing.

As we suggested in the Introduction, there are two types of magnetoreception in weak MFs: specific and nonspecific ones. Specific magnetoreception is a genetically-mediated and elaborated in the course of biological evolution ability of many species to perceive the slight geomagnetic variations of the order of 10 nT and *utilize* these in order to survive. Nonspecific magnetoreception is mostly a random response to MF changes—one that is not due to specific localized sensitive biophysical structures, is not used by organisms to survive, but having the nature of the adaptation syndrome to a stress-factor. The mechanisms of these two types of magnetoreception have a common physical basis in the interaction of the magnetic moments with the MF, but differ in the next level of biophysical/biochemical events that are initiated by peculiarities of the motion of the magnetic moments. Nonspecific magnetoreception could be of fundamental importance in terms of health risks caused by a chronic EM exposure of humans and biosphere.

Two dimensionless quantities presumably control the occurrence of non-specific effects of the molecular nature produced by uniaxial ac/dc MFs, which has been discussed repeatedly in the literature. This is the ratio of the ac MF frequency to the cyclotron frequency of the proposed target–particle, and the ratio of the amplitude of the MF to its permanent component. These quantities may affect the occurrence of magnetic effects only along with other physico-chemical and physiological factors. These factors are difficult to control; therefore, the occurrence of non-specific effects on the whole is less predictable. However, such effects are more likely to occur under the following conditions

Ω/γH∼1 (19)

h/H∼1 (20)

which is in agreement with the experimental data and theoretical models. In the first equation *γ* is the gyromagnetic factor. In the case of a target–particle with charge *q* and mass *M*, this is *γ* = *q*/*Mc*, where *c* is the speed of light. Indeed, there are no other combinations of target parameters with the dimension of frequency besides *γH*. This equation requires one to select the ac MF frequency approximately proportionate to the dc MF magnitude. The second equation requires to choose ac MF amplitude approximately equal to the dc MF magnitude.

As follows from this article, in addition to the relations Eqs (19) and (20), the dimensionless quantity

γHτ∼1 (21)

controls the occurrence of the HMF effects, see Eqs (4), (6), (12) and(13), and Figs 4A, 5 and 6. In order for an HMF effect to arise, this equation requires the MF to be reduced to the order of 1/*γτ*. Eq (21) is another general ratio in magnetobiology that defines the thermal relaxation time of the MF targets to be a parameter directly measurable in the experiment, as shown in Section “Universal physical mechanism.”

All these relations Eqs (19)–(21) give the scope for interpretation of a variety of experimental curves. All what is needed is a fast and inexpensive experimental model of nonspecific magnetoreception with an enhanced level of reproducibility. Perhaps, such a model is bacterial or plant cell cultures in conjunction with the parallel sequencing in gene expression that would allow one to choose some responding genes for subsequent quantitative PCR.

## Conclusion

In this review, we analyzed (i) a hundred original experimental works on the effects of HMF and (ii) several theoretical models in terms of their ability to explain and predict observations. The result of the analysis are the following statements.

Nonspecific magnetoreception different from the magnetic navigation abilities of animals is of fundamental importance and has its own biophysical mechanisms.

Biological effects of HMF are directly related to nonspecific magnetoreception and are the most promising tool in establishing its nature.

Despite the apparent existence of the nonspecific biological effect of HMF, no regular connection could be found of the magnitude of this effect with the physical conditions of the experiments. The correlations of the HMF effect with the field magnitude, type and duration of exposure, and the MF inhomogeneity are close to zero. This suggests that there is no general biophysical MF target that is one and the same in different organisms. We think that there is a general MF target only at the primary phyisical level, in the form of a magnetic moment of yet unknown nature. A log-normal distribution of the HMFs used says more about socially defined and random selection of the HMF values rather than about their purposeful choice.

In addition to well-known approaches to magnetoreception—RP and magnetite-based mechanisms—original results are presented. They examine these and other mechanisms regarding their explanatory capabilities of how HMF magnetoreception is possible. Mechanisms that have a predictive power in relation to the HMF effect are: primary universal physical mechanism, gyroscopic mechanism, and possibly that based on water protons. The protonic mechanism is not ruled out, but not yet developed enough for constructive predictions. A method that allows one to select an actual mechanism among presented ones is based on using different modes of magnetic exposure applied to the same organism.

The most attractive model of HMF magnetoreception is the universal physical model that is capable of predicting the type of MF-dependencies for different types of magnetic exposure, and is universally applicable to precessing magnetic moments of any nature. The model allows one to interpret the experimental data on the effects of magnetic vacuum and extract information about the MF target—its gyromagnetic ratio and the thermal relaxation time. The combination of these values is closely related to the physical nature of the MF primary target and can greatly assist in its identification. Thus, there suggested a new methodological space in magnetic biology—finding the physical characteristics of the target by comparing the responses to the different modes of magnetic exposure in the same biological system.

The critical level of constant MF, or 1/*γτ*, is inversely proportional to the product of the gyromagnetic factor and the thermal relaxation time of the magnetic moment of the target. In this or in smaller fields, an effect of the MF on molecular targets is possible.

The fact that a magnetically modifiable biological response strongly depends on the parameters of the target magnetic moments as well as the physical exposure conditions strongly argues that the experiments should be undertaken, when ever possible, under conditions that reduce the magnetic variables, i.e., under HMF conditions. This may in fact be the only way for future identification of the primary physical MF target in organisms.

## Supporting information

S1 Table Full table on the effects of hypomagnetic field. (XLSX)

S1 References The list of missed literature. (PDF)
