# Supplementary material for: Biological effects of the hypomagnetic field: An analytical review of experiments and theories
Source: PLoS One. 2017 Jun 27;12(6):e0179340. doi: 10.1371/journal.pone.0179340 (PMC5487043; doi:10.1371/journal.pone.0179340)
Supplement: S1 References — (PDF) [file pone.0179340.s002.pdf]

S2 References. The list of missed literature

The list of articles that have not been included in Table 1 of “Biological effects of the hypomagnetic field: an analytical review of experiments and theories” by V.N. Binhi and F.S. Prato

1. Adamkiewicz V. W., Bassous C., Morency D., Lorrain P., Lepage J. L. Magnetic response in cultures of Streptococcus mutans ATCC-27607. Experimental Biology, 46(3):127-132, 1987.
2. Afonina V. M., Chernyshev V. B., Yarovenko S. A. Effect of shielding from electromagnetic field on life span of drosophila. In: Afonina V. M., ed. Effect of Natural and Weak Artificial Magnetic Fields on Biological Objects. Belgorad, Belgorad Press. P.83-84, 1973. [Афонина В. М., Чернышев В. Б., Яровенко С. А. Влияние экранирования от ЭМП на продолжительность жизни мух-дрозофил. В кн.: Влияние естественных и слабых искусственных магнитных полей на биологические объекты. Белгород, 1973, с. 83-85.]
3. Alferov O. A. and Kuznetsova T. V. Effect of an attenuated geomagnetic field on Escherichia coli UV ray resistance. Kosm. Biol. Aviakosm. Med., 15(4):57-58, 1981.
4. Babych V. I. The characteristics of tissue lipid peroxidation in the internal organs and the lipid metabolic indices of the blood plasma in a low geomagnetic field. Fiziol Zh, 41(5-6): 44-49, 1995 [Укр. Физиологичний журнал].
5. Babych V. I. The characteristics of tissue lipid peroxidation of the internal organs in anaphylaxis under the action of a hypo- or hypermagnetic field. Fiziol Zh, 42(5-6): 66-71, 1996 [Укр. Физиологичний журнал].
6. Babych V. I. Serotonin metabolism under the action of a low geomagnetic field. Fiziol Zh, 42(1-2):79-82, 1996 [Укр. Физиологичний журнал].
7. Beischer D. E. Human tolerance to magnetic fields. Astronautics, 7(3):24-48, 1962.
8. Becker R. O. The biological effects of magnetic fields - a survey. Med. Electron. Biol. Eng., 1(3):293-303, 1963.
9. Becker R. O. … New York J. Med. 63, 2215, 1963.
10. Becker G. Zur magnetfeld-orientierung von dipteran. J. Comparative Physiol. A. 51(2):135–150, 1965.
11. Becker G. Influence of magnetic, electric and gravity fields on termite activity. Material and organism, 3:407-418, 1976.
12. Belyavskaya N. A. Changes in plastid ultrastructure in pea meristem cells exposed to magnetic fields with conditionally zero magnetic intensity. Ukrainian Bot. J., 37(1):81-82, 1981 (in Ukrainian).
13. Bin Zhang et al. Exposure to hypomagnetic field space for multiple generations causes amnesia in Drosophila melanogaster. Chinese Biological Abstracts, 20:60-61, 2006 (In Chinese).
14. Borodin Yu. I., Letiagin A. Yu. Reaction of circadian rhythms of the lymphoid system to deep screening from geomagnetic fields of the earth. Bulletin of Experimental Biology and Medicine, 109(2):191-193, 1990. [Бородин Ю. И., Летягин А. Ю. Реакция циркадных ритмов лимфоидной системы на глубокое экранирование от геомагнитного поля Земли. Бюллетень экспериментальной биологии и медицины, 109(2):191-193, 1990.]
15. Bregvadze V. S. Influence of hypomagnetic field on the septal and raphe nuclei neuronal and glial cells in tissue culture. Radiation Studies, 5(11):181-187, 2000.
16. Ding H.-M., Mo W.-C., Fu J.-P., Hu J.-H., Liu Y., Hua X. The hematopoietic system responses to one-month continuous hypomagnetic field exposure in adult mice. Advances in Modern Biomedicine, 14:5001-5004, 2014 (Article in Chinese).

17. Edmiston J. Effect of exclusion of the earth's magnetic field on the germination and growth of seeds of white mustard Sinapis alba. Biochemie und Physiologie der Pflanzen, 167(1), 97–100, 1975.
18. Fomicheva V. M., Govorun R. D., Danilov V. I., Belyavskaya N. A., Budyashova S. Y., Zaslavsky V.A. The effect of natural fluctuations of the geomagnetic field on the initial stages of high plant ontogeny. Dopovidi Akademii Nauk Ukrainskoi RSR, Seriya B Geologichni Khimichni Ta Biologichni Nauki, 10:74-77, 1990 (In Ukrainian).
19. Greene A. E. and Halpern M. H. Response of tissue culture cells to low magnetic fields. Aerospace Med. 37(3): 251-253 (1966).
20. Halpern M. H. Effect of reproducible magnetic fields on the growth of cells in culture, NASA CR 75121, Washington: NASA, 1966.
21. Halpern M. H., Dyke J. H. Very low magnetic fields: Biological effects and their implications for space exploration. Aerospace Medicine, 37:201 (or 281), 1966.
22. Ioale P., Teyssedre A. Pigeon homing: effects of magnetic disturbances before release on initial orientation. Ethol Ecol Evol, 1:65-80, 1989.
23. Kazimov A. P. About effect of screening of natural electromagnetic fields on contents of green pigments in bean leaves. In: Flora and Vegetation of Kazakhstan. Alma-Ata, (10):53-58, 1984 (in Russian).
24. Kaznacheev V. P., Mikhailova V. P.., Ivanova M. P., Zaitsev Y. A. and Kharina N. I. Peculiarities of the growth and behaviour of the cell monolayer in the hypomagnetic field. , in Biophysical and Clinical Aspects of Heliobiology, ed. M. N. Gnevishev, Nauka, Leningrad, 1989, pp. 189-195. (or in Problems of Cosmic Biology, 65)
25. Khodorkovskii V., Polonnikov R. Studies of hyperweak magnetic field reception in fish. In Fish Behavior, p. 72. Kaliningrad State Univ., 1971. [Ходорковский В. А., Полонников Р. И. Исследование рецепции крайне малых магнитных полей в рыбах. В: Вопросы поведения рыб. Калининград: КГУ, 1971, С. 72.]
26. Kolmakov V. M., Kulikov V. Yu., Evstropov A. N., Voronin A. Yu. Evaluation of the influence of deep geomagnetic impacting on growth rate and sensitivity to antibiotics in Escherichia coli. Zh Mikrobiol Epidemiol Immunobiol 3:68-70, 2002. (In Russian)
27. Kopanev V. I., Efimenko G. D., Shakula A. V. Biological effect of a hypogeomagnetic environment on an organism. Izvestiya Akademii Nauk SSSR Seriya Biologicheskaya, 6(3):289-298, 1979. [Копанев В.И., Ефименко Г.Д., Шакула А.В. О биологическом действии на организм гипомагнитной среды. Известия Академии Наук СССР. Серия биология, (3):342-354, 1979.]
28. Kursevich, N. V., Travkin, M. P. Effects of magnetic fields with different intensities on some enzymes activities in barley seedlings. In: Effects of Natural and Weak Artificial Magnetic Fields on Biological Objects. Belgorod Teachers Training College Publishing Co., Belgorod, Russia, pp. 102-104, 1973. (in Russian).
29. Kursevich, N.V., Travkin, M.P. Effects of weak magnetic fields on root growth and the respiration intensity in barley seedlings. In: Effects of Natural and Weak Artificial Magnetic Fields on Biological Objects. Belgorod Teachers Training College Publishing Co., Belgorod, Russia, pp. 104-106, 1973. (in Russian).
30. Lebedev S. I., Baranskiy P. I, Litvinenko L. G, Shiyan L. T. Barley growth in superweak magnetic field. Electronic Treatment of Materials, (3):71-73, 1977.
31. Levina R. V., Smirnov R. V., Olimpienko T. S. Effect of a hypogeomagnetic field on warm-blooded animals. Kosm Biol Aviakosm Med, 1989, 23(1): 45-47 (in Russian)
32. Li J., Wu Q., Wang Q., et al. Magnetic free field space and albino of golden hamster. Chinese Science Abstracts, 5(6):781-783, 1999. [In Chinese]
33. Liu L., Wang J., Jin H., Zhang Y., Zhao L., Zheng Q., Chen W. and Zhao S. Biological effect of magnetic field-free space on wheat. Acta Agricuturae Nucleatae Sinica (Henong Xuebao), 16(1):2-7, 2002.

34. Marhold, S., Burda, H., Kreilos, I. & Wiltschko, W. Magnetic orientation in common mole-rats from Zambia. In Orientation and navigation - birds, humans and other animals. Paper no. 5. Oxford, UK: Royal Institute of Navigation, 1997.
35. Miro L., Deltour G., Pfister A., Kaiser R., Grandpierre R. Biological effect of hypomagnetic environment. La Presse thermale et climatique. 107(1):32-34, 1970. Article in French.
36. Neamtu S., Truta Z., Boldizsar E., Pop C. V. L., Morariu V. V. Cell motility in zero magnetic field, Studia Universitatis Babes-Bolyai, Physica, (4): 697-700, 2005.
37. Nechiporenko, G. A., Dobrovolski, M. V., Novitsky, I. Y. The effect of weak permanent magnetic field on the content of main cations in onion organs and in radish plants of basic magnetically-oriented types. In: Plant Under Environmental Stress. Publishing House of Peoples' Friendship University of Russia, Moscow, pp. 205-206, 1973.
38. Nepomniashchikh L. M., Lushnikova E. L., Klinnikova M. G., et al. Tissue and intracellular reorganization of the mouse myocardium induced by the hypogeomagnetic field. Biull Eksp Biol Med, 124(10):455-459, 1997.
39. Novitskaya, G. V., Tulinova, E. A., Kocheshkova, T. K., Novitsky, I. Yu. The effect of weak permanent magnetic field on cotyledon emergence and neutral lipid content in 5-day-old radish seedlings. In: Plant under Environmental Stress. Publishing House of Peoples' Friendship University of Russia, Moscow, pp. 212-213, 2001.
40. Sandodze V. Influence of hypomagnetic medium on proliferation activity of the hippocampal fascia dentata and ammon's horn suprafimbrial cells in early- and late ontogenesis. Radiation Studies, 5(9): 188-196, 2000.
41. Selitskii G. V., Karlov V. A., Sorokina N. D. The influence of hypogeomagnetic field on bioelectric activity of the brain in epilepsy. Zh Nevrol Psikhiatr Im S S Korsakova, 99(4):48-50, 1999.
42. Shibib K., Brock M., Gosztony G. The geomagnetic field: A factor in cellular interactions. Neurosci Res. 9(4):225-235, 1987.
43. Shiyan L. T. Study on the ecological significance of geomagnetic fields as an example of plants. Sci. Trans. Kursk Teacher's Training College, 191:82-83, 1978 (in Russian).
44. Shust I. V., Kostinik I. M. Effect of a strong constant magnetic field and a hypomagnetic surrounding medium on histochemical indices of albino rat liver. Kosm. Biol. Aviakosm. Med. 9(6):19-25, 1975. [Шуст И. В., Костиник И. М. Влияние сильного постоянного МП и гипомагнитной окружающей среды на гистохимические показатели печени белых крыс. Космическая биология и авиакосмическая медицина, 9(6):19-28, 1975.]
45. Shust I. V., Kostinik I. M. Reaction of the animal adrenal cortex to the action of a strong, constant magnetic field and to a hypomagnetic environment. Probl Endokrinol (Mosk.) 22(2):86-92, 1976 (article in Russian). [Шуст И. В., Костиник И. М. Реакции коры надпочечников животных на воздействие сильного постоянного МП и гипомагнитной среды. Проблемы эндокринологии, XXII(2):86-91, 1976.]
46. Sosunov, A. V., Golubchak, B. A., Semkin, V. Y. and Melnikov, A. V. Observation of some biological processes in shielded spaces. In: Sanitary Assessment of Magnetic Fields, Academic Press, Moscow, 144-146, 1972.
47. Tomita-Yokotani K., Yamashita M., Yanagisawa M., Hashimoto H., Nakamura T., Hasegawa K. Effect of a low magnetic condition on growth movement in plants. Biol. Sci. Space, 14:238-239, 2000.
48. Tomita-Yokotani K., Yanagisawa M., Yamashita M., Fukuda T., Suzuki T., Yamada K., Nakamura T., Hasegawa K. Phototropism under microgravity or low magnetic condition. Space Utiliz. Res., 17:119-122, 2001.
49. Travkin M. P., Antipova N. M. Effect of reduced magnetic field on development and fecundity of drosophilia melanogaster. In: Afonina V. M., ed. Effect of Natural and Weak Artificial Magnetic Fields on Biological Objects. Belgorad, Belgorad Press. P.82, 1973.

50. Trofimov A. V., Sevostyanova E. V. Dynamics of blood values in experimental geomagnetic deprivation (in vitro) reflects biotropic effects of natural physical factors during early human ontogeny. Bull Exp Biol Med, 146(7):109-113, 2008. [Трофимов А.В., Севостьянова Е.В. Динамика параметров крови при экспериментальной геомагнитной депривации (in vitro) как отражение биотропных воздействий природных физических факторов в раннем онтогенезе человека. Бюллетень экспериментальной биологии и медицины, 146(7):109, 2008.]
51. Truta Z., Neamtu S., Morariu V. V. Zero magnetic field influence on in vitro human spermatozoa cells behavior. Rom. J. Biophysics, 15:73-77, 2005.
52. Truta Z., Lerintiu S., Garlovanu M., Morariu V. V. Zero magnetic field influence on male reproductive cells progressive motility distribution. Studia Universitatis Babes-Bolyai - Studia Physica, (1):39-43, 2006.
53. Van Dyke J. H., Halpern M. H. Observations on selected life processes in null magnetic fields. Anatomical record, 151:480-481, 1965.
54. Yamashita M., Yamashita A., Yamada M. Three-dimensional (3-D) clinostat and its operational characteristics. Biol. Sci. Space, 11:112-118, 1997.
55. Yu Q., Liu L., Huang B., et al. Selection of high quality male sterility line of Indica rice by field free magnetic space inducement and its character. Journal of Nuclear Agricultural Sciences, 20(6):497-499, 2006.
56. Yu Q., Liu L., Xu G., et al. Study on mutagenic effects of magnetic field free space on dry rice seed. Acta Agricuturae Nucleatae Sinica, 16(3):139-143, 2002.
57. Zhang Yu., Tang F., Zhang H., et al. Breeding of new variety nongjing no. 1 of medicago sativa l by magnetic field free space. Acta Agricuturae Nucleatae Sinica, 21(1):34-37, 2006.
58. Анисимов С. В., Бакастов С. С., Гапеев А. К., Копылов А. И., Крылова И. Н., Масленникова Т. С., Абашина Т. Н., Арискина Е. В., Вайнштейн М. Б., Сузина Н. Е. Экспериментальное исследование влияния магнитного поля на удельную скорость ассимиляции карбонатного углерода у бактерий Pseudomonas fluorescens. Биология внутренних вод, (2):21-28, 2005.
59. Ачкасова Ю. Н. Метаболизм и скорость размножения микроорганизмов, развивающихся при экранировании электрических и магнитных полей. В кн.: Влияние магнитных полей на биологические объекты. Харьков, 1973. С.51-53.
60. Ачкасова Ю. Н., Владимирский Б. М. Реакция микроорганизмов на воздействие магнитного поля с частотой в диапазоне КПК типа Pc 2. В кн.: Влияние естественных и слабых искусственных магнитных полей на биологические объекты. Белгород, 1973, с. 12-16.
61. Воронин А. Ю., Куликов В. Ю., Гайдуль К. В кн.: Регуляция пролиферативной активности стволовой кроветворной клетки геомагнитными полями низкой напряженности. Бюллетень СО РАМН, (3):93-97, 2001.
62. Галантюк С. И. Гистохимические исследования печени и селезенки белых крыс, подвергнутых воздействию ПМП. В кн.: Общие закономерности морфогенеза и регенерации. Тернополь, 1975, с. 54-62.
63. Глейзер С. И., Ходорковский В. А. Экспериментальное определение геомагнитной рецепции у Европейского угря. Доклады АН СССР, 201(4):964-967, 1971.
64. Денисенкова И. В., Пискунова Г. М., Чемерис Н. К. Стимулированная локомоторная активность планарии Dugesia tigrina в естественном магнитном поле и при его компенсации. Вестник новых медицинских технологий, 4(4):56-60, 1997.
65. Забродина Д. В. Действие магнитного поля очень низкой напряженности на свертывающую систему крови. В кн.: Исследования по геомагнетизму, аэрономии и физике Солнца. Вып. 17. М.: Наука, 1971, с. 68-71.
66. Козяева Е. А., Куликов В. Ю., Бут В. Д., Шот Ю. А., Даниличев М. С. Активность плазменных факторов свертывания крови при воздействии различных магнитных флуктуаций. Медицина и образование в Сибири, (3), 2011.

67. Козяева Е. А., Орумбаева С. К., Бахтина И. А. и др. Прооксидантный и антиоксидантный потенциал мононуклеаров крови человека в условиях ослабленного геомагнитного поля. Экология человека, (4, прил 1):109-111, 2006.
68. Козяева Е. А., Куликов В. Ю., Колмаков В. М. и др. Влияние условий ослабленного геомагнитного поля на АДФ-зависимую агрегацию тромбоцитов у практически здоровых доноров. Вестник МНИИКА, (10):80-85, 2003.
69. Конашев М. Б. , Азизова Г. Н. , Горшков Э. С. Влияние магнитного поля на микромицеты. Микология и фитопатология, 27(1):42-45, 1993.
70. Кривова Н. А., Труханов К. А., Замощина Т. А. и др. Повышение агрессивности крыс при экспозиции в условиях гипогеомагнитного поля. Авиакосмическая и экологическая медицина, 42(6/1):30-32, 2008.
71. Куликов В. Ю., Козяева Е. А., Сорокин О. В. Влияние слабых экологических факторов на систему гемостаза и реактивность клеток эффекторов воспаления в эксперименте. Мир науки, культуры, образования, 28(3):320-326, 2011.
72. Куликов В. Ю., Сорокин О. В., Орумбаева С. К., Козяева Е. А., Абрамцова А. В. Прооксидантный и антиоксидатный потенциал мононуклеаров в условиях ослабленного геомагнитного поля. Сибирское медицинское обозрение, 70(4):11-16, 2011.
73. Нахильницкая З. Н., Мастрюкова В. М., Адрианова Л. А. и др. Реакция организма на воздействие «нулевого» магнитного поля. Космическая биология и медицина, 12(2):74-81, 1978.
74. Панасюк Е. Н., Бабич В. И., Лыч О. С., Кит В. И. Действие ослабленного магниного поля Земли на систему свертывания крови. Космическая биология и авиакосмическая медицина, 25(3):59-60, 1991.
75. Сванидзе И. К., Сандодзе В. Я., Дидимова Е. В. Чхиквадзе Т. И., Портной В. Н., Раздольский А. С. Изучение влияния гипо- и гипермагнитных полей на двигательную активность реснитчатого аппарата эпендимных клеток. Радиационная биология, радиоэкология, 34(1):100-105, 1994.
76. Сосунов А. В., Головецкий А. С, Маник Ю. С. О солнечно-земных отношениях в патогенезе сердечно-сосудистых заболеваний человека. В кн.: Климат и сердечно-сосудистая патология. М.: Наука, 1966, с. 10-11.
77. Стрижижевский А. Д. Некоторые закономерности физиологической регенерации тканей млекопитающих в условиях воздействия сильных магнитных полей. Проблемы космической биологии, 1978, т. 37, с. 31-50.
78. Сушков Ф. В. Эквивалентность некоторых реакций культуры клеток ткани на увеличение и уменьшение магнитного поля. Физико-математические и биологические проблемы действия электромагнитных полей и ионизации воздуха, т.2. М.: Наука, 1975. С. 112-113.
79. Травкин В. В. Влияние МП на природные популяции. В кн.: Реакция биологических систем на магнитные поля. М.: Наука, 1978, с. 127-130.
80. Трипузов А. М., Мамыкин В. Н. Действие постоянного магнитного поля на лизогенный штамм кишечной палочки к-12. В кн.: Материалы II Всесоюзного совещания по изучению влияния магнитных полей на биологические объекты. М.: Наука, 1969, с. 228-232.
81. Холодов Ю. А. Реакции организма животных и человека на магнитные поля. В кн.: Реакция биологических систем на МП. М.: Наука, 19786, с. 13-18.
82. Чуваев П. П. Влияние сверхслабого постоянного магнитного поля на ткани корней и проростков и на некоторые микроорганизмы. В кн.: Материалы II Всесоюзного симпозиума по изучению влияния магнитных полей на биологические объекты. М.: Наука, 1969, с. 252-256.
83. Шакула А. В., Черняков Г. М. Влияние ГГМП на активность некоторых ферментов головного мозга. Гигиена и санитария, (9):105, 1981.
